# Three Sliding Probes Placed on Forelimb Skin for Proprioceptive Feedback Differentially yet Complementarily Contribute to Hand Gesture Detection and Object-Size Discrimination

**DOI:** 10.1007/s10439-023-03434-4

**Published:** 2024-01-21

**Authors:** İsmail Devecioğlu, Ertuğrul Karakulak

**Affiliations:** 1 Biomedical Engineering Department, Çorlu Faculty of Engineering, Tekirdağ Namık Kemal University, Tekirdağ, Turkey; 2 Department of Biomedical Device Technologies, Vocational School of Technical Sciences, Tekirdağ Namık Kemal University, Tekirdağ, Turkey

**Keywords:** Proprioception, Prosthesis, Sensory substitution, Sensory feedback, Touch

## Abstract

The purpose was to assess the effectiveness of three sliding tactile probes placed on the forelimb skin to provide proprioceptive feedback for the detection of hand gestures and discrimination of object size. Tactile contactors representing the first three fingers were driven along the proximodistal axis by linear servo motors. Twenty healthy subjects were involved in the gesture detection test, with 10 of them also participating in the object-size discrimination task. Motors were controlled by computer in the first four sessions of the gesture detection experiment, while the fifth session utilized a sensorized glove. Both the volar and dorsal sides of the forearm were examined. In the object-size discrimination experiment, the method was exclusively assessed on the volar surface under four distinct feedback conditions, including all fingers and each finger separately. The psychophysical data were further analyzed using a structural equation model (SEM) to evaluate the specific contributions of each individual contactor. Subjects consistently outperformed the chance level in detecting gestures. Performance improved up to the third session, with better results obtained on the volar side. The performances were similar in the fourth and fifth sessions. The just noticeable difference for achieving a 75% discrimination accuracy was found to be 2.90 mm of movement on the skin. SEM analysis indicated that the contactor for the index finger had the lowest importance in gesture detection, while it played a more significant role in object-size discrimination. However, all fingers were found to be significant predictors of subjects' responses in both experiments, except for the thumb, which was deemed insignificant in object-size discrimination. The study highlights the importance of considering the partial contribution of each degree of freedom in a sensory feedback system, especially concerning the task, when designing such systems.

**Supplementary Information:**

The online version contains supplementary material available at 10.1007/s10439-023-03434-4.

## Introduction

We all highly depend on interactions with our environment in our daily living activities such as feeding, cleaning, driving, socializing, etc. This interaction includes collecting information via our sensory organs and actions generally executed by our muscles. Our actions are usually flawless thanks to the closed-loop circuit between the sensory and motor systems. However, this closed-loop circuit may get disturbed by a trauma or a disease affecting one or both sensory and motor systems. Amputation resulting from a chronic disease or trauma is among the most frequently encountered conditions where both the sensory and motor organs are lost. People with limb amputation suffer from physical, social, psychological, and even economic problems [1, 2]. Providing an esthetical prosthesis has been the initial solution for amputees [3]. Although these prostheses only provide improvements in personal appearance anxiety, independence in daily living activities is still a challenging issue. To provide amputees with a substitution for a real limb, extensive studies have been conducted on motorized prostheses. There are commercialized prostheses that encode the indented movements from electromyography (EMG) signals (i.e., Michelangelo by Ottobock Healthcare GmbH, Germany, and i-Limb^®^ by Össur, Iceland). However, the users must watch the movements performed by these devices, because they lack the essential sensory signals, such as touch and proprioception, from the device. Therefore, the sensory-motor loop is partially completed by the vision. Yet an intact sensory-motor loop is essential for motor planning [4] and the embodiment of the limbs [5]. Furthermore, in a survey study, Lewis [6] reported that amputees would prefer sensory feedback from their prosthesis in the form of vibration, electrical stimulation, pressure, or temperature instead of watching or listening to their prosthesis.

To satisfy the needs of amputees and decrease the rate of prosthesis abandonment, a huge effort has been put into somatosensory neuroprosthesis (see [7] for a detailed view of approaches). Different invasive and noninvasive methods have been proposed to substitute touch and proprioception [8–18]. Invasive methods have some drawbacks, such as their invasive nature, encoding information through electrical stimulation, and long-term condition of implanted electrodes that must be tackled to reach the end-user [8–11]. On the other hand, noninvasive methods, which mostly depend on sensory substitution, come forward with their convenience of use [12–18]. Sensory substitution proposes feedback sensory information related to a missing sensation or sensory organ via an intact sensation or sensory organ by recording the information for the target organ. Most of the sensory substitution methods are proposed to provide tactile information because detecting the initial contact with an object and controlling the force exerted on the object is important for the correct handling of the object. Nevertheless, proprioception is also critical in the planning of the movement and correcting for errors during the movement. Therefore, if we want the prosthesis to substitute a lost limb rather than being just a tool [19], both tactile and proprioceptive information must feedback to the user.

In the literature, there are studies where the movement of joints was feedback to users as discrete events using vibrotactile or electrotactile stimulation. Discretization may be as simple as the flexion and extension of the joints [18, 20] or may include different levels of joint movement (e.g., the level of stimulation indicates some intermediate angles) [21, 22]. Proportional mapping between the joint angle and the level of stimulation has also been demonstrated in the literature. In this case, the amplitude of the feedback stimulus (i.e., electrical [23], the amount of skin stretch [15, 17, 24], or the location of the stimulus (e.g., location of a probe indenting the skin [16, 25, 26]) varies with the angle of the joint. Akhtar et al. [15] proposed to stretch the skin of the forearm proportional to the angle of a robotic finger. Similarly, Battaglia et al. [17] presented a device that stretched the skin of the upper arm in the mediolateral axis. Wheeler et al. [24] tested to provide proprioceptive feedback using torsion of the skin. With these three methods, subjects could utilize the provided feedback in a joint angle adjustment task or in a hand gesture detection task. Rossi et al. [16] proposed a device consisting of a linear actuator equipped with a wheel to feedback the movement of a joint to the user. The wheel was contacting the skin and moved on the proximodistal axis proportional to the movement of a robotic hand. The subjects could discriminate the sizes of different objects grasped by the robotic arm. In a previous study, we showed that healthy humans can detect the angle of a virtual joint with a similar device which consisted of a linear servo motor equipped with a contactor sliding on the forearm skin [12]. In 2020, we also presented our preliminary findings on how successfully the healthy subjects can discriminate between 6 different grip gestures with the help of three actuators of the same kind used in our previous study [25]. In a more recent study, Cha et al. [26] tested the same method by changing the mapping between the fingers and the actuators (equipped with contactor wheels as in Rossi et al.) in a myoelectric robotic hand control task. They used three linear actuators for the forefinger, middle, and ring fingers (one actuator for each; assuming the ring and little fingers moved together) and a rotational servo indenting the skin to signal the movement of the thumb. The subjects successfully discriminated 10 grip gestures performed by the robotic hand and feedback by the actuators positioned on the upper arm. Although the contactors were positioned proportional to the position of fingers in the later two studies, the actual feedback used in the tests was discrete; the contactors were positioned at predefined locations on the skin based on the presented hand gesture.

As the capacity of prosthetic hands to perform various gestures improves, research on tactile sensory substitution focuses on delivering relevant feedback more comprehensively within the limited skin area on the stump of amputees. Nevertheless, achieving this goal still requires the utilization of multiple feedback channels. Considering the existing literature on sensory substitution for proprioceptive feedback and recognizing the intricacies of hand movements, it is intriguing to explore the extent to which the provided feedback signals are both relevant and comprehensive for the user. In this study, we tested a tactile sensory substitution method, which we proposed in 2020 [12, 25], for providing proprioceptive information from multiple fingers and analyzed the contribution of each feedback channel to the performance of subjects in two psychophysical tasks. The method uses three rigid tactile contactors which are slid on the forearm skin by linear servo motors in our case. Positions of contactors were adjusted based on the angles of three fingers (thumb, forefinger, and middle finger). The control signals (i.e., finger movements) were generated by a custom program or a sensorized glove worn by one of the researchers. The method was tested on healthy volunteers in two tasks: hand gesture recognition and object size discrimination. We also analyzed the improvement of psychophysical performance over time, and contribution of each feedback channel on the responses of subjects. Results indicate that the position of fingers and the diameter of objects can be estimated with the feedback provided by each channel of which importance may vary depending on the task but stay similar across subjects.

## Materials and Methods

### Subjects

Twenty healthy subjects (10 males and 10 females; age: 21.8 ± 1.6 years) were included in the study. All were right handed and had no history of disease or trauma affecting the sense of touch. Experimental procedures were explained to all subjects, and informed consent was asked. Experiments were approved by Tekirdağ Namık Kemal University Ethical Committee for Non-invasive Clinical Research. Experiments were conducted in compliance with the principles of the Declaration of Helsinki.

### Sensory Substitution Method

**Fig. 1:**
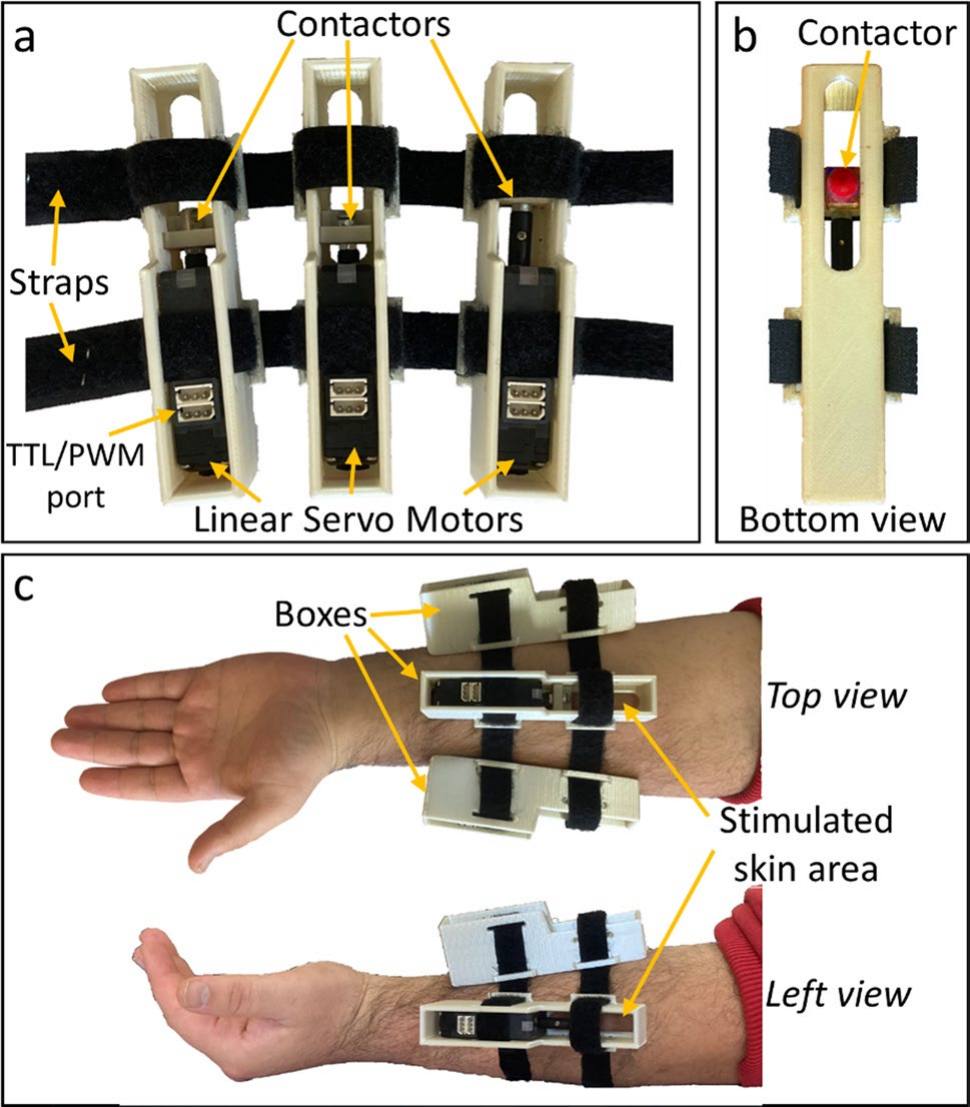
The sensory substitution device (**a** and **b**) and its volar montage on the forearm (**c**)

The device used for testing the proposed sensory substitution method consisted of three linear servo motors (mightyZap D7-6PT-3; IR Robot, South Korea) each equipped with a tactile contactor (Fig. 1). One servo motor was used for each following: the thumb, the forefinger, and the middle finger. Contactors were 3D printed with PLA and had a round tip with a diameter of 4 mm. The surfaces of the contactors were smoothed by sanding and dying with acrylic dye. The servo motors were placed in separate boxes and mounted on the subject’s left forearm with Velcro straps. Contactors were protruding ~ 2 mm from the opening on the bottom of the boxes. The motors were controlled by either a custom MATLAB (2016a; The Mathworks, USA) script or a sensorized glove worn by a researcher. The movement distance of the contactor probes was 26 mm.

**Fig. 2:**
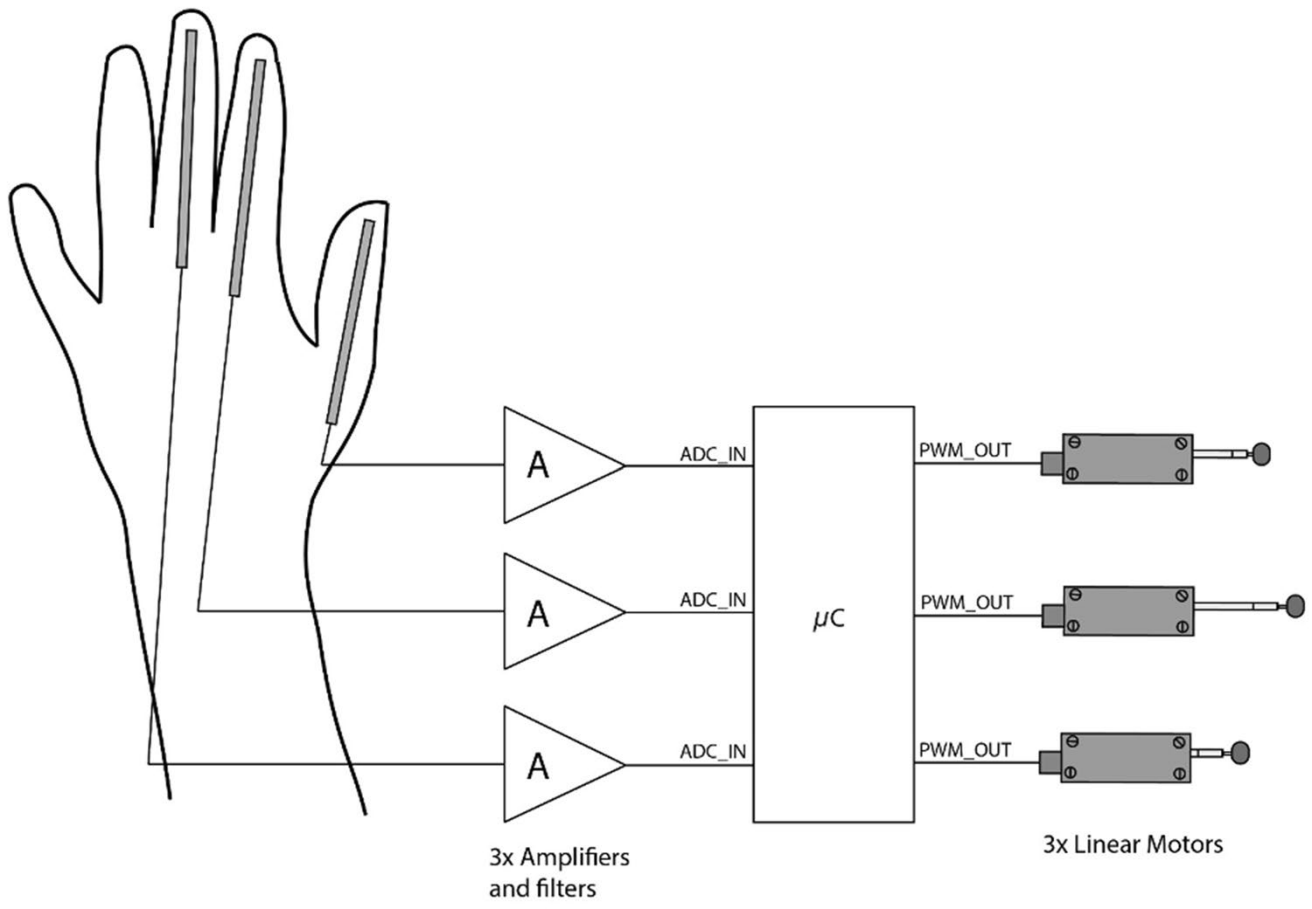
The sensorized glove and servo motor control circuit block diagram

The device was tested on both the volar and dorsal surfaces of the forearm. Each motor was positioned on the forearm so that the motor representing the thumb was on the lateral side of the arm, the motor representing the middle finger was on the most medial side of the volar/dorsal surface, and the motor representing the forefinger was in between them. A PC-USB interface (mightyZap IR-USB01; IR Robot, South Korea) was used to control servo motors when MATLAB script was used. We built an analog circuit to measure finger positions from flexible sensors (SEN-08606; Spectra Symbol, UT, USA) on the glove (Fig. 2). Wheatstone bridge coupled with a differential amplifier was used to measure sensor signals and signals were low-pass filtered (*f*_c_ = 1.2 Hz). A microcontroller (STM32F103c8t6; ST Microelectronics, Sweden) was used to acquire signals and control servo motors via pulse width modulated (PWM) signals (pulse duration: 0.9–2.1 ms, *f* = 250 Hz). The PWM outputs were calibrated with hand-open and hand-closed conditions at the beginning of each session where the motors were controlled with the glove. The delay between hand movements and motor movement was 272.7 ± 13.6 ms as measured at 90% of the maximum displacement of the servo motor.

A linear mapping between sensor data and positions of tactile contactors was used in gesture detection tests because the tested hand gestures require the movement of contactors to specific locations on the skin. So, the mapping was not a significant factor affecting the psychophysical performance. On the other hand, the output of the flex sensors and the analog circuit was non-linear. To improve the discriminability of the object sizes in experiment II, we used a third-degree polynomial function for equidistantly mapping five object sizes as well as full extension and flexion of the hand on the skin (Eq. (1), Fig. S1).

1 $${t}_{{\text{PWM}}{-}{\text{Duty}}}=\sum_{i=0}^{3}{a}_{i}{q}_{{\text{ADC}}}^{i}.$$

In Eq. (1), *q*_ADC_ is the measured sensor signal on the analog-to-digital converter of the microcontroller (resolution: 12-bits), ${t}_{{\text{PWM}}-{\text{Duty}}}$ is the duty period of the PWM signal in milliseconds (0.9–2.1 ms) that is used to drive servo motors, and ${a}_{i}$ are the coefficients fitted to the calibration data (*R*^*2*^ > 0.99). For calibration purposes, the output of the sensor circuit was measured one minute after an object was grasped (or hand opened/closed) so that the sensor output was stabilized. Measurements were repeated five times.

### Experiment I: Detecting Hand Gesture

**Fig. 3:**
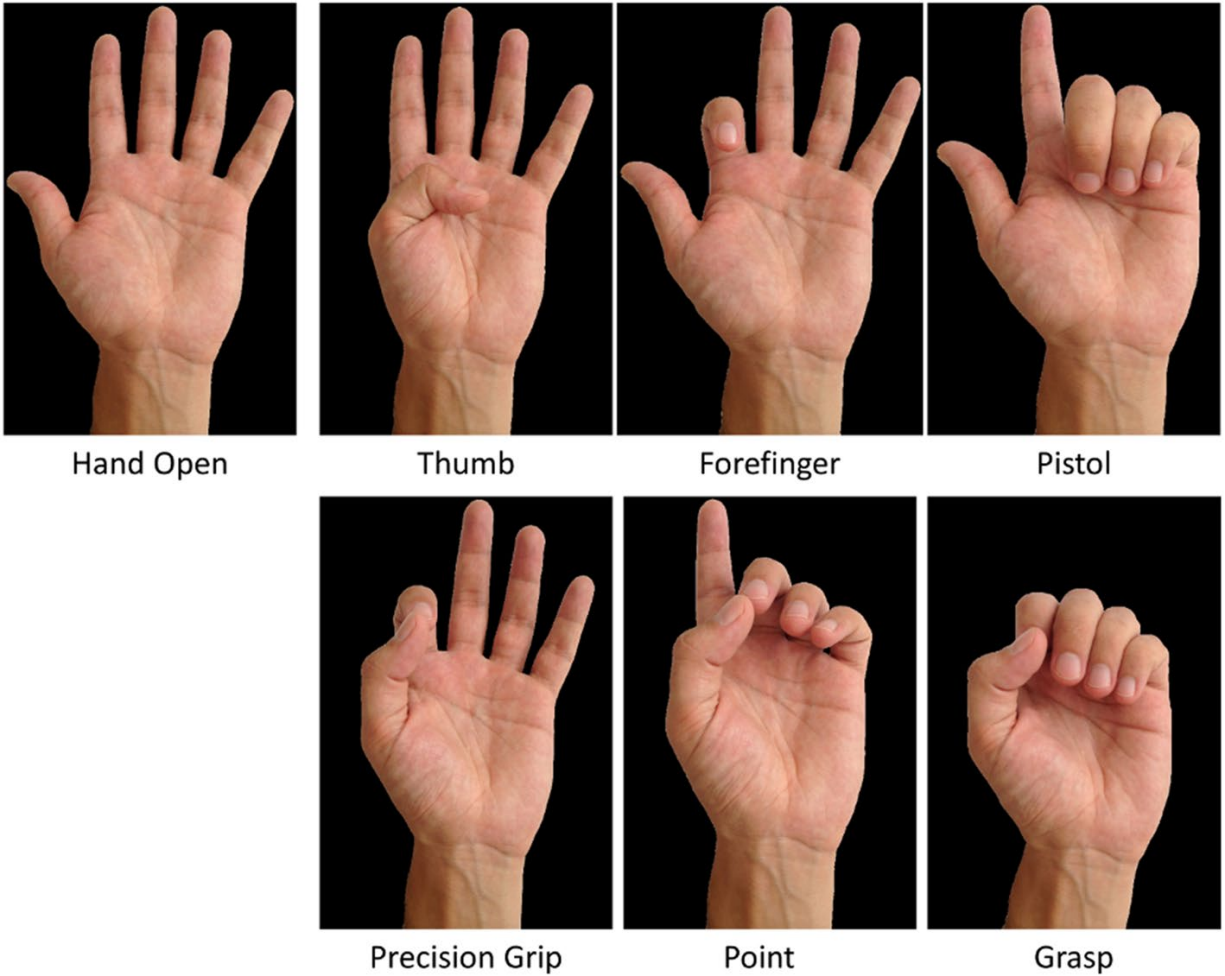
Hand gestures used in the Experiment I

We tested the performance of subjects in detecting six hand gestures (Fig. 3). The sensory substitution device was mounted on either the volar or dorsal side of the subject’s left forearm and covered with a box. The subject sat in front of a PC monitor and listened to white noise to prevent auditory cues from servo motors. He/she used his/her right hand to respond via a mouse during experiments. Five sessions were performed at approximately 1-week intervals. Each session consisted of three phases: training, demo test, and test. The training phase consisted of 12 trials. In each trial, the subject was presented with an image of one hand gesture randomly selected among six hand gestures on the monitor. Meanwhile, the related contactor positions were also presented via the sensory substitution device. Each hand gesture was presented two times, and the subject was asked to actively observe both the image on the monitor and contactor movements and positions on the skin. The demo test phase consisted of 18 trials. In each trial, the subject was presented with images of six hand gestures on the screen, while the contactor positions related to one randomly selected hand gesture were also presented on the skin. The subject was asked to select the hand gesture associated with the contactor positions. The response period was 5 seconds. If the subject selected the correct hand gesture on the screen, then it was highlighted with a green border. Otherwise, the correct hand gesture was highlighted with a green border, and the choice of the subject was highlighted with a red border. If the subject did not respond within 5 seconds, then the correct option was highlighted with a green border. The trials in which the subject did not respond were not repeated. The test phase consisted of 60 trials where each hand gesture was presented ten times in random order. In each trial, the subject was presented with images of six hand gestures on the screen, and a contactor pattern related to one randomly selected hand gesture. The subject had to select one hand gesture with no time limit. After the subject responded, no feedback was given about the correctness of the choice, and a new trial was initiated. Each trial started with an open-hand image shown on the monitor and contactor positions at open-hand representation. The order of the presented hand gestures, the responses of the subject, and the error matrix were recorded at the end of each session.

In experiments with the dorsal montage of the sensory substitution device, an open hand was represented with the servo motors fully extruded, and the contactors were close to the elbow. When a finger was closing, the related contactor was moved distally. We inversed the mapping for the volar montage. We have chosen this strategy based on our pilot experiments. In addition, the contactors were moved as if they were controlled by the muscles that control the fingers with this strategy [12, 16]. The order of volar and dorsal montage was randomized across sessions.

In the first four sessions, the servos were controlled by the custom MATLAB script. The contactor movement speed was 36 mm/s. In the fifth session, the motors were controlled by a researcher who wore the sensorized glove on his left hand. The same researcher presented the hand gesture for every subject. The phases were the same as in previous sessions. The selected hand gesture in each trial was shown to the researcher on a second PC monitor. The researcher and the subject sat facing each other and were separated by a curtain so that the monitor and the hand of the researcher were out of the subject’s vision. Twenty subjects were tested in the first four sessions, while 10 subjects (5 males and 5 females) who had completed the first four sessions were tested in the fifth session.

### Experiment II: Object Size Discrimination

We tested the performance of 10 subjects (those included in the fifth session of the first experiment) in discriminating the size of two objects consecutively held by the researcher. Two-interval forced-choice paradigm with the method of constant stimuli was adopted [27]. The sensory substitution device was mounted on the volar surface of the subject’s left forearm and covered with a box. We have chosen the volar surface because subjects performed slightly better with this montage. The researcher wore the sensorized glove on his left hand and sat across from the subject. A PC monitor was used to instruct the researcher about the stimulus intervals and size of objects to be held. The same researcher wore the glove and presented the stimuli for each subject. A custom-made response box was used to instruct the subject about the stimulus and response intervals. Stimulus intervals were indicated by red and green light-emitting diodes (LEDs), and response interval was indicated by the yellow LED. The subject had to respond with either the red or green button located under the relevant LEDs.

Five cylinders with different diameters (1 cm, 3 cm, 5 cm, 7 cm, and 9 cm) were used. This set of object sizes provided a proper distribution of grasp openness starting from full extension to full grasp. Based on our pilot experiments, the intermediate cylinder (5 cm) was chosen as the standard stimulus, and all cylinders were compared to it. Each cylinder was tested 20 times in random order. The interval of the standard stimulus was also randomized and counterbalanced across trials. The experiment was repeated in four conditions: all three servo motors were active together in a session, and each servo motor was tested in separate sessions. Correct discrimination rates were recorded and fitted with logistic distributions of the form given in Eq. (2).

2 $$P=\frac{1}{1+{{\text{e}}}^{(M-D)/S}},$$

where *P* is the probability of a comparison stimulus called bigger, *M* is the mean of the distribution, *S* is the variance of the distribution, and *D* is the diameter of the comparison stimulus.

### Statistical Analysis

Statistical analyses were done in SPSS (v18; SPSS Inc., Chicago). The Kolmogorov–Smirnov (K–S) test was used to test if variables were normally distributed or not. The mean and standard deviation are given for normally distributed data, while the mean, median, and quartiles are given for others.

In Experiment I, the average confusion matrices for volar and dorsal montages were built. The mean correct estimation rate and 95% confidence interval (CI) were calculated for each hand gesture across subjects. CIs were checked if a hand gesture was successfully detected above the chance level (1/6 or 16.7%). In addition, the accuracy, precision, sensitivity, and specificity were calculated based on error matrices and averaged across subjects for each session. A linear mixed-effects model analysis was performed to see if these parameters were affected by the first four sessions, skin site, hand gesture, sex, and/or pairwise combinations of any factors. The random effects due to subjects and sessions were considered in the model. Levels of factors yielding a significant effect were compared in post hoc analysis with Bonferroni correction. The fifth session, which was conducted with the sensorized glove, was compared to the fourth session with paired t test.

In Experiment II, the K–S test was used to test if psychometric curves recorded in different conditions were similar. The estimations and 95% confidence intervals for *M* and *S* in Eq. (2) were explicated by comparing them between conditions. The Kruskal–Wallis test was used to compare the just noticeable difference (JND) between test conditions.

### Structural Equation Model Analysis

We conducted structural equation modeling (SEM) analyses to assess the contribution of each feedback channel (i.e., each tactile stimulator) on the perception that gave rise to the specific responses in each trial. We preferred SEM over regression analysis because SEM let us consider latent variables (i.e., sensations elicited by each contactor and overall perception) that we cannot observe directly. In addition, SEM is less strict about the assumptions for the distribution of data, and this is important as our data (i.e., applied stimulus and responses) are not continuous and represented as categorical and/or ordinal dummy variables.

**Fig. 4:**
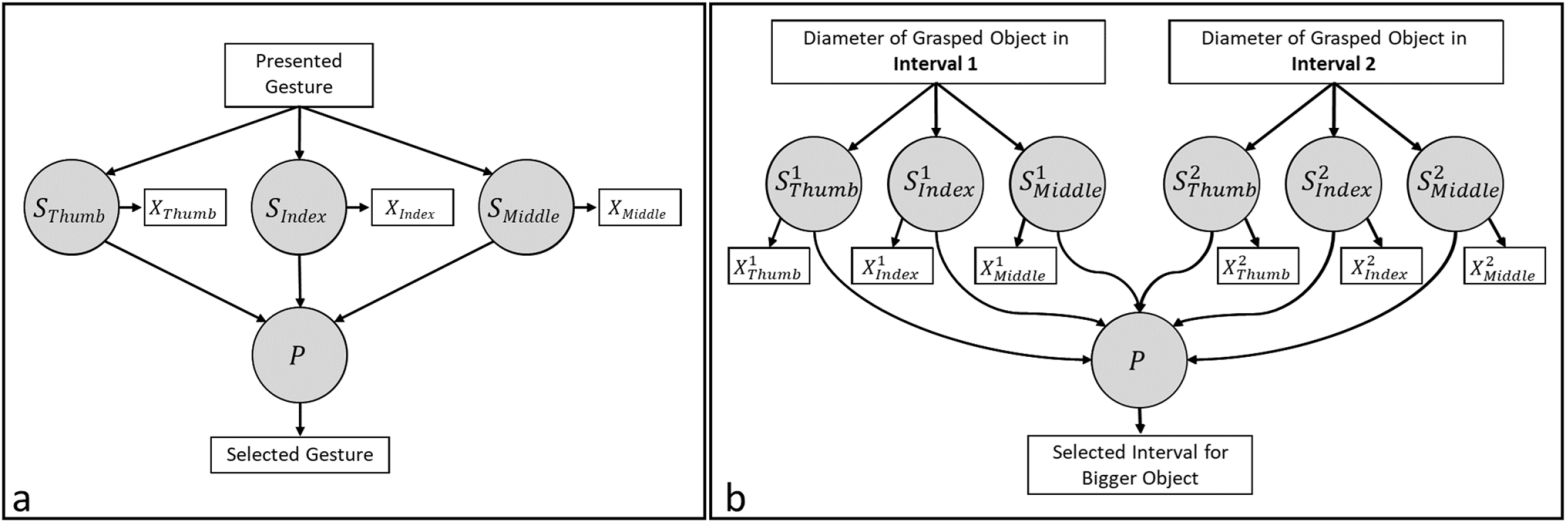
Structural equation models built for **a** Experiment I and **b** Experiment II. Rectangles represent the observed variables while circles represent latent (unobservable) variables.

We built one model for each experiment (Fig. 4a, b), but the models have a similar information flow. Each contactor’s position/movement on the skin elicits a sensation [i.e., *S*_*f*_, *f*: finger (Thumb, Index, Middle)] which then shapes the overall perception (*P*) resulting in a specific response (e.g., selecting a gesture in Experiment I or selecting an interval in Experiment II). Experiment II is different from Experiment I in means of the number of stimuli presented in a trial. For this reason, we included two subsets of variables representing the sensations elicited in two intervals. We might use different *P* variables (probably with a covariance effect) for each interval as well, but we have only one measurement related to the perception (a measure showing the direction of the difference in perceptions in each interval). Therefore, we found the current version of the model more consistent with the experimental task.

For the model of Experiment I, hand gestures were represented with numbers (1: thumb, 2: forefinger, 3: pistol, 4: precision grip, 5: point, and 6: grasp; “Presented Gesture” in Fig. 4a). The state of contactors (i.e., *X*_*f*_ = 0 if contracted and *X*_*f*_ = 1 if extended) for each gesture was used as measures of the related sensations (i.e., *S*_*f*_). The response was presented with the number of the selected gesture (“Selected Gesture” in Fig. 4a). Two approaches of analysis were used for Experiment I. First, we performed a leave-one-out analysis in which we trained the model by excluding one subject and tested the trained model with the excluded subject. For this purpose, we used the data from the first four sessions of Experiment I. Second, we trained the model using all subjects’ data from the first four sessions and tested the trained model with the data from the fifth session in which the system was controlled by the experimenter wearing the sensorized glove. The model generated outputs on a continuous scale in the test phase. For this reason, the output of the model was rounded to the nearest integer and compared with the response of the subject.

The diameters of the grasped objects in each interval are provided to the model for Experiment II (“Diameter of Grasped Object in Interval 1/2” in Fig. 4b). The foreseen position of contactors (*X*^*i*^_f_, *i*: interval, *f*: finger) based on Eq. (1) for each grasped object were used as measures of the related sensations (*S*^*i*^_*f*_). The response was presented as − 1 for the first interval and + 1 for the second interval (“Selected Interval for Bigger Object” in Fig. 4b). We used the ± 1 convention to improve the model’s performance. This is convenient because the decision variable (*P*) takes the difference of magnitudes of sensations from both intervals (see “Results” section). Since the model generated outputs on a continuous scale, the sign of the output value was compared to the response of the subject.

Performance was evaluated as the percentage of correct estimations by the model for the subject’s response. We recorded estimates for associations between variables in the models, the correct rate, and some information criteria (the Bayesian information criterion, BIC, and the Akaike information criterion, AIC) from each fold of training and testing. We used *semopy* to build and solve models in Python [28].

## Results

### Experiment I: Detecting Hand Gesture

#### Psychophysical Experiments

**Fig. 5:**
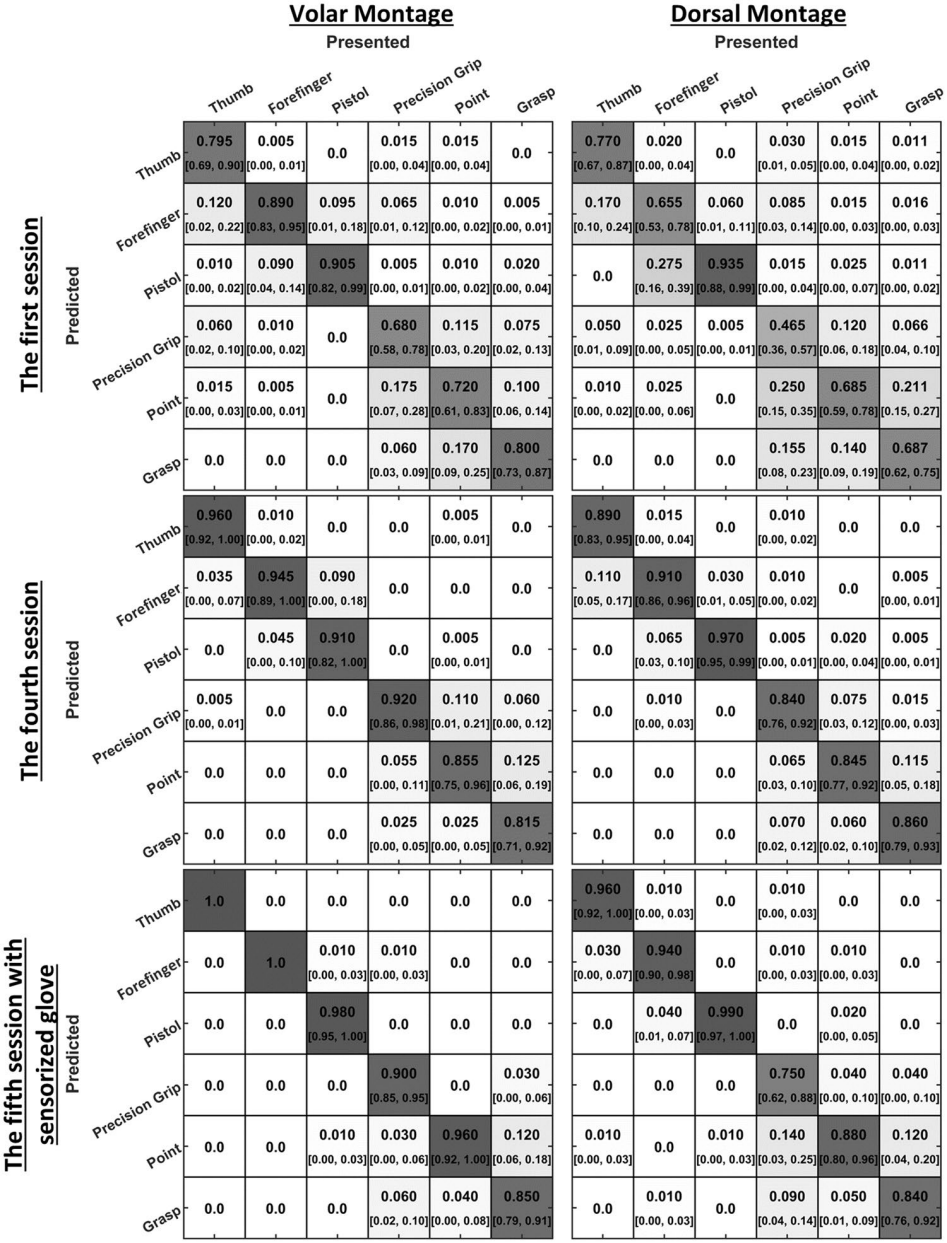
Confusion matrices for detecting hand gestures. In each square, the average correct rates and 95% confidence intervals are given. If the confidence interval involves the chance level (1/6), then that means a systematic error. Systemic errors decreased with practice (1st vs 4th sessions) and further reduced in the fifth session where the actuators were controlled with a sensorized glove.

Each hand gesture was successfully detected above the chance level starting from the very first session (Fig. 5). If the confusion matrices in the first and the fourth sessions are compared, it is seen that the correct rates increased, and error rates decreased by session. The average correct rate (aCR) in the first session was 79.83% in volar montage and 69.95% in dorsal montage, while aCR in the fourth session was 90.08% in volar montage and 88.58% in the dorsal montage. Nonetheless, although systemic errors were reduced in the fourth session compared to the first session, still there existed some. In the volar montage, the pistol gesture was confused with the forefinger gesture 9% of the time. The pointing gesture was confused with the precision grip gesture 11% of the time. The grasp gesture was confused with the point gesture 12.5% of the time. In the fourth session of the dorsal montage, the thumb gesture was confused with the forefinger gesture 11.0% of the time. The grasp gesture was confused with the point gesture 11.5% of the time.

In the fifth sessions of volar and dorsal montages, aCRs were 94.8% and 89.3%, respectively (Fig. 5). Like the first four sessions, hand gestures with one-finger movement (volar aCR = 99.3%, dorsal aCR = 96.3%) were better detected than others (volar aCR = 90.3%, dorsal aCR = 82.3%). The grasp gesture was confused with the pointing gesture for both volar and dorsal montages. Only in the dorsal montage, the precision grip gesture was confused with the point gesture.

**Fig. 6:**
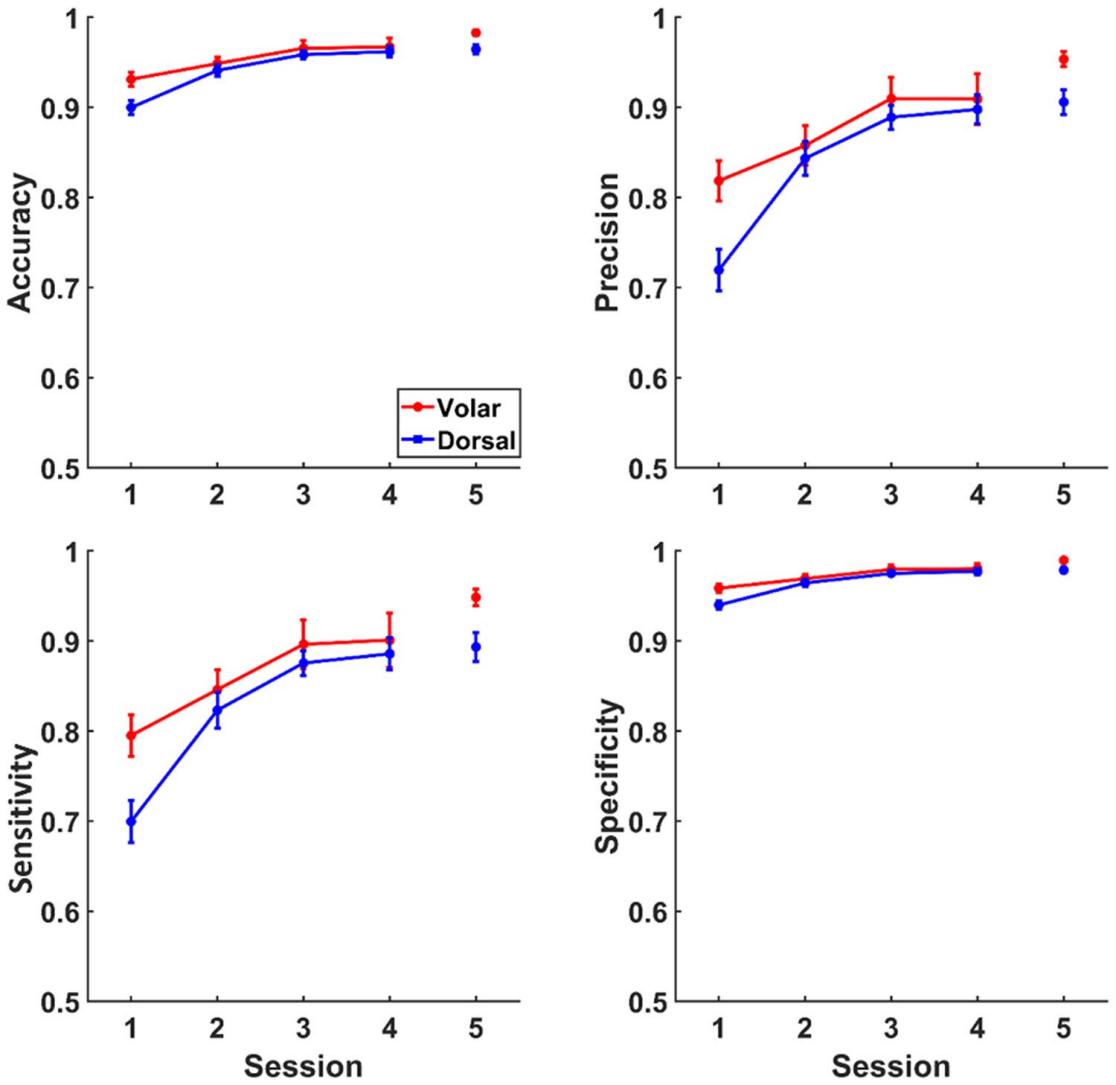
Change of performance scores across the sessions. All performance scores increased with practice while they were generally higher in the volar condition. The fifth session was performed with sensorized glove. Data points show the average of subjects, and error bars show the standard error.

Average accuracy, precision, sensitivity, and specificity were > 0.85 in volar montage and > 0.80 in dorsal montage (Fig. 6). Therefore, the subjects could successfully and reliably detect a hand gesture and differentiate it from others. The linear mixed-effects model analysis revealed that these scores were significantly affected by the session (*p* < 0.001 for all scores). All scores increased with the first two sessions, and then, reached the plateau (Fig. 6). The skin site also affected the scores (*p* < 0.001 for all scores). The subjects performed significantly better with volar montage compared to dorsal montage. An interaction was detected between the session and the skin site (*p* < 0.05). The interaction was mainly due to the difference between the scores for volar and dorsal montages in the first session (Fig. 6). All scores were also affected by hand gestures (*p* < 0.001); hand gestures with one-finger movement were detected better than others. The interaction between the session and the hand gesture was also significant (*p* < 0.05). The scores for each hand gesture increased at different rates depending on the degrees of freedom in the hand gesture. Sex and other interactions did not significantly affect any score (*p* > 0.05). In the fifth session where the experiments were done with the sensorized glove, all performance scores were higher than in the previous sessions (Fig. 6).

#### SEM Analysis

The model estimated the subjects’ responses with an accuracy of 86.22 ± 7.86% for the volar condition and 71.11 ± 8.11% for the dorsal condition (Table S1). Meanwhile, the model’s accuracy in selecting the correct gesture was always perfect. It should be noted that the model yielded very similar association estimations for each tested subject for both volar and dorsal conditions. In both conditions, the feedback path for the middle finger had the highest association strength at all levels (e.g., “*Presented Gesture*” to ${S}_{Middle}$, and ${S}_{Middle}$ to *P*) whereas the index finger had the lowest. These estimations were always found to be significant (*p* < 0.001) except in one case with the dorsal data (training with Subjects 1–19, and testing with the Subject 20) where estimated associations between ${S}_{f}$ and *P* were not significant (*p* > 0.99). Nevertheless, the accuracy of the model for this case was high (82.43%).

**Fig. 7:**
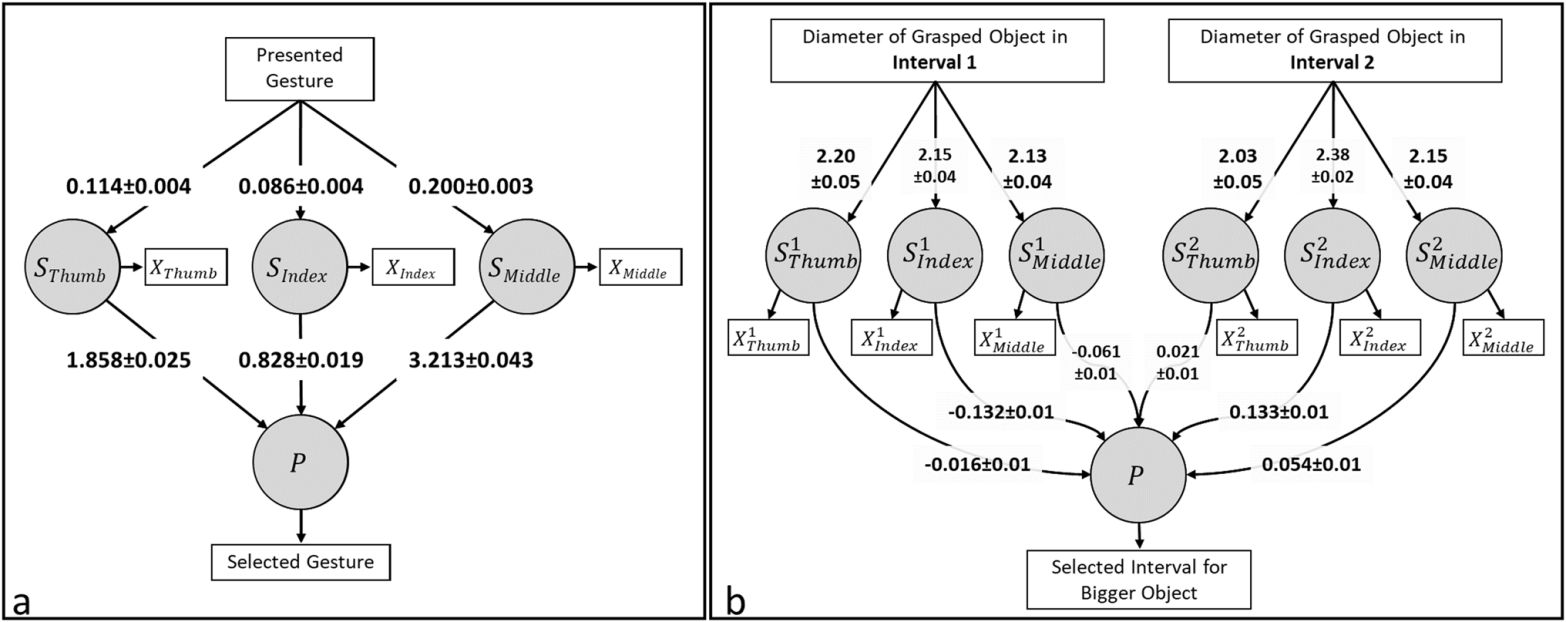
Estimated associations between model components for **a** Experiment I and **b** Experiment II. Predictive value of each sensory channel changed with behavioral task. Nevertheless, they were all significant predictors of subjects’ responses except the thumb in Experiment II.

When the model was trained with all subjects’ data from the same condition (volar/dorsal), estimated associations were like those observed in the leave-one-out analysis (Table S1 and Fig. 7a). The accuracy of the model in estimating the subjects’ responses was 94.67 ± 2.17% for the volar condition and 76.67 ± 7.99% for the dorsal condition.

### Experiment II: Object Size Discrimination

#### Psychophysical Tests

**Fig. 8:**
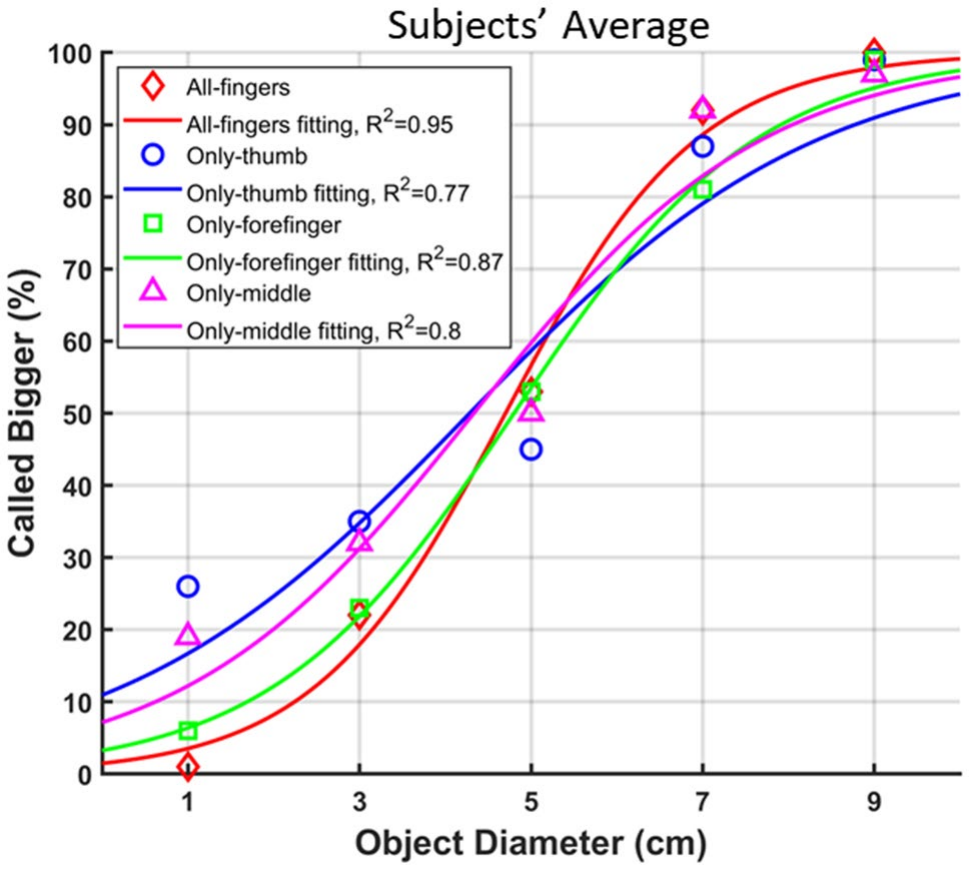
Object size discrimination performance averaged across subjects. The data for each condition were fitted with Eq. (2), and *R*^*2*^ values of the fits are given in the legend. Data points show the average of subjects and solid lines show the fitted curve. The steeper the curve, the better the discrimination performance is. When feedback about all fingers was presented, subjects discriminated the size of objects better than when information about a single finger was presented.

The subjects’ discrimination rate increased as the diameters of two objects got farther apart in all conditions (Figs. 8 and S2). The median correct discrimination was 100% [85.0-100%] (mean = 92.2%) when four comparison stimuli (diameters: 1 cm, 3 cm, 7 cm, and 9 cm) were pooled in the all-fingers condition. Subjects almost perfectly discriminated the 1- and 9-cm objects from the standard stimulus (1-cm object: median = 100% [96.7%, 100%], mean = 99.0%; 9-cm object: median = 100% [100%, 100%], mean = 100%). The median accuracy was 80.0% [69.9%, 86.1%] (mean = 78.0%) for the 3-cm object and 90.0% [86.4-97.6%] (mean = 92.0%) for the 7-cm object. The psychometric data from single-finger conditions were not different from all-fingers condition (K–S test: only-thumb *p* = 0.24, only-forefinger *p* > 099, only-middle finger *p* = 0.68). The best fit with Eq. (2) was obtained for the all-fingers condition (*R*^*2*^ = 0.95), while the worst fit was for the only-thumb condition (*R*^*2*^ = 0.77). The 95% confidence interval of the mean (*M* in Eq. (1)) of the fitted distribution included the diameter of the standard stimulus (5 cm) only for the only-forefinger condition. It was wider for the only-thumb and only-middle finger conditions compared to the other two conditions (all-fingers: *M* = 4.70 [4.48, 4.91] cm; only-thumb: *M* = 4.28 [3.78, 4.78] cm; only-forefinger: *M* = 4.80 [4.45, 5.15] cm; only-middle finger: *M* = 4.33 [3.88, 4.78] cm). The variance (*S* in Eq. (1)) of the distribution was the smallest for the all-fingers condition and the largest for the only-thumb condition (all-fingers: *S* = 1.12 [0.92, 1.31]; only-thumb: *S* = 2.04 [1.54, 2.55]; only-forefinger: *S* = 1.42 [1.10, 1.74]; only-middle finger *S* = 1.69 [1.26, 2.12]). For the given standard object radius, we calculated the 50% JND in object diameter as 1.16 ± 0.43 cm in the all-fingers condition, 1.96 ± 0.87 cm in the only-thumb condition, 1.40 ± 0.62 cm in the only-forefinger condition, and 1.77 ± 0.82 cm in the only-middle finger condition. Regarding the distance between contactor positions with object sizes 1 cm and 9 cm (~ 20 mm), JND for contactor movement was found as 2.90 ± 1.08 mm in the all-fingers condition, 4.90 ± 2.17 mm in the only-thumb condition, 3.50 ± 1.56 mm in the only-forefinger condition, and 4.44 ± 2.05 mm in the only-middle finger condition. The differences were not statistically significant (*p* = 0.08).

#### SEM Analysis

The model estimated the subjects’ responses with an accuracy of 86.44 ± 3.49%, while it perfectly estimated the interval in which a bigger object was presented (Table S2). Although the estimated associations at the first level of the model (e.g., “Diameter of Grasped Object in Interval 1/2” to ${S}_{f}^{i}$) were very similar to each other, the associations between ${S}_{f}^{i}$ and *P* were different between fingers and stimulus intervals (Fig. 7b). Feedback for each finger had almost the same associative strength with *P,* but the direction of association was opposite between stimulus intervals. Estimated associations at the first level were always found to be significant (*p* < 0.001). Estimations for the paths from ${S}_{{\text{Index}}}^{1/2}$ and ${S}_{{\text{Middle}}}^{1/2}$ were also mostly significant (*p* < 0.05 for all, except for one case where *p* = 0.058; Table S2). On the other hand, estimations for thumb were mostly found to be insignificant (*p* > 0.068 for all, except for one case where *p* = 0.037; Table S2).

## Discussion

In a previous study, we demonstrated the effectiveness of sensory feedback via sliding probes on the forearm skin in a joint angle adjustment task [12]. In this study, we tested the same method in hand gesture detection and object-size discrimination experiments with healthy subjects. The main differences between the two setups are the number of actuators used and the distance traveled by a contactor on the skin. The results showed that subjects successfully detected six hand gestures with the tested sensory substitution method for finger proprioception. Although some systematical errors were observed, the performance increased with experience. In size discrimination tests, subjects better discriminate the comparison object from the standard one as the sizes get farther apart. Although the results are not as good as those expected in the intact sensory condition, they are comparable to the findings in the literature and may promise a sensory and motor improvement in the use of prostheses. Nevertheless, the role of each actuator may significantly vary depending on the task which should be considered in the design of sensory substitution methods with multiple degrees of freedom.

### Detecting Hand Gesture

The average correct rates in the fourth and fifth sessions were higher than that reported for the skin stretch method in the literature (> 88.5% in this study vs. 88.00% in [15]). Nonetheless, these scores were lower than that reported by Cha et al. (97.43% in [26] vs. 94.8% in the volar montage of the fifth session of the current study). This is most likely due to the differences in the montage configuration and the skin area tested in the studies (see below).

The results showed that it was harder to identify moving probes when more than one was moving. As anticipated, identifying a single probe moving on the skin was easier, as fewer elements could interfere with perception. However, when multiple probes were in motion, the sensations from different probes probably interfered with one another. The main confusion was about the movement of the contactor related to the forefinger; either the movement of this contactor could not be discriminated from others, or the subjects felt like it was the moving contactor although it was not. Although we tried to maximize the distance between contactors, the subjects struggled to discriminate between the movements of contactors since the tactile acuity on the forearm skin is not as good as it is on the hand and fingers [29]. Cha et al. [26] tested a very similar method on the upper arm of the healthy subjects where the contactor wheels were placed wide apart from each other. They did not report any systematical error in a grasp gesture detection task. It is possible to test the same method on the upper arm with the device that we use (even by increasing the number of hand gestures). Although we intuitively foresee that the results will be very similar to Cha et al., we should note that we do not propose a haptic device in this study, rather we tested the method on the forearm in specific (maybe, in stricter) configurations that might be used with a prosthesis (see “Possible Strategies for Prosthetic Implementation” section). In addition, we adopted a rule for the relationship between the movement of a contactor and the movement of a finger: it was as if the contactor was controlled by the muscles controlling the finger. Utilizing the information from sensory substitution devices presents a significant cognitive load with or without controlling a myoelectric prosthetic hand. Distributing the contactors around the forearm may confuse the user in means of how they move based on the fingers or may require different mapping rules (i.e., movement direction of the contactors in relation to the movement of fingers) which will further increase the cognitive load while reducing the benefits. However, subjects' performance in detecting hand gestures with multiple degrees of freedom improved with practice, albeit at a slower rate compared to hand gestures involving a single-finger movement. Therefore, maximizing the distance between actuators and commencing training with single-finger movements, which facilitates an easier association of perceptions with finger movements, may enhance overall long-term performance.

The subjects learned to detect and differentiate between six hand gestures over sessions as shown by the increase in accuracy, precision, specificity, and sensitivity. The initially low performance in the first two sessions may be attributed to the significance and sensitivity of touch on the forelimbs. However, studies suggest that tactile detection or discrimination ability may improve with conditions such as blindness [30], sensory-attentional training [31, 32], and occupational experience [33]. Performance improvement with practice has been also observed in other applications, such as using robotic hands and tactile feedback [34, 35]. In our study, subjects who were unaccustomed to using their forelimb skin for such purposes initially faced challenges in discriminating the details of probe movements on the skin. However, as they engaged with the task and focused on the feedback signal, their ability to detect and discriminate informative aspects of the signals increased over sessions. Nevertheless, the average accuracy eventually plateaued at around 90%. This plateau may be attributed to inherent noise in sensation, as physiological conditions such as innervation density and cortical representation remain consistent after 4 weeks of practice. While further practice in healthy subjects may not significantly enhance physiological conditions, extensive training could potentially yield notable improvements in cortical representation for amputees, owing to residual brain plasticity. In literature, it has been shown that neural networks in the brain areas related to body image and sensory-motor areas change with extensive use of hand-held tools, so we get better at using these tools [19, 36–38]. Therefore, with extensive training, the subjects’ performance may further improve, at least until they do not systemically confuse the movements of contactors. Moreover, implementing extensive training programs for individuals who will use prostheses with sensory feedback may facilitate a more rapid adaptation to the device. Yet further longitudinal studies are required to test this hypothesis.

The average accuracies in the fifth session where the contactors were controlled by the sensorized glove were generally better than those in the fourth session. The first four sessions were idealized so that the motors’ control signals were not affected by any sensory noise and the positions of the fingers were ideally detected. In the fifth session, however, noise is raised due to the movements of the flex sensors. Although we low-pass filtered the sensor signals, the PWM signals had some noise resulting in mechanical noise on the skin. The unsteady movement of the contactor probes (mostly during the movement) might excite the mechanoreceptors of the skin (i.e., hair follicles and/or other cutaneous receptors such as Merkel cells). As a result, the moving contactor was better identified.

Although accuracy, precision, specificity, and sensitivity were similar in the last sessions of volar and dorsal montages, the difference is prominent in the confusion matrices and the change across sessions. In our previous study, where we tested the same feedback technique with a single actuator, we did not observe a significant difference in performance between volar and dorsal conditions [12]. However, in this preceding study, the subjects' task was to locate the single-joint pendulum based on the contactor's position on their skin. Therefore, it is possible that perceptions from dorsal and volar skin were of similar quality when there was only one tactile contactor. In the current study, subjects presented with multiple probes moving together had better perceptive quality on volar skin than they did on dorsal skin. A significant difference in the performance is naturally expected for dorsal and volar feedback conditions due to the differences in the skin structure and sensory innervation [29]. For example, there are more hairs on the dorsal skin compared to the volar skin. In our case, the movement of hair follicles might have created a more disturbed sensation, causing a decrease in the quality of the feedback signal. Nevertheless, the subjects' performance increased and eventually reached almost the same level on volar and dorsal skin. In any case, delivering feedback signals mostly on the volar surface would aid in the adaptation period in a multiple-degree sensory feedback design. Meanwhile, males and females also differ in means of mechanical properties and sensory innervation of the skin [39]. Nonetheless, sex was not a significant factor affecting the performance which is consistent with the tactile psychophysical studies on glabrous skin [40, 41].

### Object Size Discrimination

The discrimination accuracy was higher compared to similar studies in the literature [16, 17]. The feedback through each actuator was delivered on a 26-mm line in our study. Rossi et al. [16] delivered the feedback on a 40-mm line on the forearm, but the diameters of tested objects were in a smaller range compared to ours (standard stimulus: 20 mm; comparison stimuli: 10-30 mm). On the other hand, Battaglia et al. [17] stretched the skin proportional to the movement of a robotic hand (maximum displacement of the skin: 10.5 mm). They tested only three object sizes (38.1 mm, 63.5 mm, and 76.2 mm) that are comparable to our intermediate object sizes. We could increase the distance of contactor movement so that the translation factor between the hand closure and contactor location would decrease (e.g., increasing the distance between two contactor positions related to two consecutive object sizes). As a result, the accuracy might improve for intermediate object sizes that we did not test in this study. However, the stump length after amputation varies from subject to subject, limiting the available space to deliver tactile feedback on the arm. For this reason, the design of the feedback device in means of movement of contactors should consider the available space on the stump of the subject.

Rossi et al. estimated JND for the movement of the wheel as 3.1 mm and 3.6 mm for volar and dorsal skin surfaces, respectively [16], which are comparable to what we observed in the all-fingers and forefinger conditions (2.9 mm and 3.5 mm, respectively). The statistical analysis failed to show any significant differences between feedback conditions, which might be due to the limited sample size. Nevertheless, the average performance was higher when the feedback was provided from all three fingers together. Although the results for the only-forefinger condition were close to the all-fingers condition, JND in the all-fingers condition indicates better discrimination is possible with a combination of all fingers. Therefore, the contribution of each finger to the perceived object size must be evaluated.

### Psychophysical Contribution of Each Feedback Channel

The models that we used in SEM analysis depend on the observed data from psychophysical experiments. However, we implemented some nodes representing the internal sensory processes that we cannot directly observe but know intuitively they exist. For example, we know that the somatosensory cortex has a somatotopic organization and different sets of neural structures process the information arising from different areas on the skin. Although the cortical magnification of the forearm skin is not as high as the fingers, psychophysical data show that our subjects were able to identify and distinguish movements of individual contactors. Therefore, the use of ${S}_{f}$ nodes as representations of sensations elicited by each contactor is legitimate. Another important concept with the models is that their first layers (e.g., “Presented Gesture” to ${S}_{f}$) were only presented by stimulus-related data (“Presented Gesture” and ${X}_{f}$) which were independent of the subject. Nevertheless, the model still interpreted the associations at this level based on the responses of the subject (i.e., considering the responses and associations in the next level). Therefore, the ${S}_{f}$ nodes are good representatives of the presumptive sensations that we cannot observe but are present in the model.

Interestingly, although we trained the models with the subjects’ responses, they were always able to estimate the correct response in each trial although the subject was incorrect for both experiments. This might be due to the high accuracy of subjects in the experiments (> ~ 75%). Nevertheless, this shows that the models were good representatives of psychophysical information processing for our experiments. Furthermore, the estimated associations between the state of each actuator and the subject’s response were consistent with what we discussed in the above sections. In the gesture detection task, for example, it was the forefinger that caused the major confusion in the responses. Consistently, the model yielded a low predictive value for the *sensations*^1^ due to the actuator for this finger. Nevertheless, it was still a significant predictor for the response as information from that actuator was necessary to distinguish between gestures that included movement of forefinger from those that did not. The association estimations for the object-size discrimination task were also consistent with our observations; the forefinger has the highest predictive importance while the thumb has a minor effect. Furthermore, the thumb was mostly found to be an insignificant predictor of the subject’s response. Therefore, the subjects mostly depend on the contactors for the fore and middle fingers. Although we mapped the sensor measurements for each object on equidistant locations on the skin, the information provided by the contactor representing the thumb might not be as clear as other fingers. However, the same experimenter wore the sensorized glove and handled the objects during the task. Further experiments with, for example, a robotic hand controlled by the subject via EMG would help us to clarify the situation. The effect of the distance between actuators should be further studied (see next section).

As we stated earlier, the estimated associations in the second level of the models are consistent with our observations. Even the associations in the first level of the model for gesture detection are consistent in the same manner. On the other hand, the estimated associations in the first level of the model for object-size discrimination lack an explicit consistency or a pattern that can be directly associated with the psychophysical data; they have similar values for both stimulus intervals. The reason lies behind the differences in the tasks; gesture detection had only one stimulus (interval) while object-size discrimination had two stimuli (intervals). In the former task, the evidence for a response is presented with the stimulus itself. On the other hand, in the latter task, the second stimulus must be waited before responding to accumulate sufficient evidence. For this reason, the predictive importance of sensations from any actuator is not clear in the first layer of the model until all evidence comes together in the second layer.

It is also interesting that the model yielded almost the same associative values for each subject. Therefore, for the given tasks in this work, the contribution of each feedback channel depended on the task while their merits from the subject’s perspective were similar. Such SEM analysis along with the improvement strategies mentioned in the following section may help optimize the placement of actuators to maximize the performance. However, further experiments with varying task requirements and sensory feedback methods with different degrees of freedom would be beneficial to understand which aspects of a feedback method depend on the task and/or the subject.

### Possible Strategies for Prosthetic Implementation

Although the proposed method is promising to convey proprioceptive information as shown in this paper and the literature [16, 26], the main challenge is how this method can be implemented in a motorized prosthesis. The devices used to provide tactile stimuli are bulky to be placed in a prosthetic socket. That is why, therefore, we did not propose a sensory substitution device in this work, but we rather tested the method itself. Indeed, we believe that the method can be implemented in a prosthesis only if the actuators are miniaturized. The actuators used here and in Cha [26] double their length in full extension compared to the retracted state. Yet the size of the actuator should not change, as in Rossi [16]. Miniaturization has two aspects: (i) vacant area in a prosthetic socket and (ii) power requirements. If the available surface area on a stump is considered, electrodes for EMG recording and the contactors for providing tactile feedback should be strategically located. The placement of EMG electrodes is the priority as it will affect the signal quality. Then, the contactors and actuators should be placed properly in the vacant area, so that their movement should minimally interfere with the EMG signals and the sensations elicited by other actuators. This issue gets more challenging as the size of the stump significantly varies from subject to subject. Therefore, different strategies should be adapted depending on the level of amputation. For example, if the stump is big then a socket including the feedback system can be used. On the other hand, if the stump after a transradial amputation is short and it is not feasible to place the feedback system in the socket, then the feedback system may be designed as an accessory device attached to the upper arm. If the amputation is transhumeral and the stump is short, then an accessory feedback device may be designed for the chest. From the power requirements aspect, we should consider the increase in the energy demand due to the electromechanical actuators. Thankfully, since the probes glide across the skin’s surface in the proposed method, the actuators do not require much torque to move the probes. Therefore, miniature linear actuators incorporating, for instance, brushless DC motors can be used.

We acknowledge that the use of rigid probes rubbing on the skin may not be feasible in long-term use. To improve the user's comfort, a layer of thin clothing can be placed on the area of stimulation. However, the clothing itself would interfere with the sense of touch and may reduce the perceived contrast between the contactor position and surrounding skin. On the other hand, contactor wheels as in Rossi [16] and Cha [26] may serve better in terms of subject comfort. However, the size of the wheel and the maximum distance that it travels would be of importance in terms of the quality of the perception. In addition, the contactor wheels used in these studies had smooth surfaces. A patterned or textured surface may increase the detectability and/or discriminability of the location of the contactor on the skin. It is also possible to employ materials with low friction coefficient or plates with adjustable friction level as tactile contactors [42]. In the latter case, additional information (i.e., rigidity of object or force at fingertip) can be encoded in friction level of the contactor.

### Limitations and Future Work

The tactile feedback provided in Experiment I can be assumed to be discrete; the contactors are moved between two predefined points. Nevertheless, the perception of movement probably contributes to identifying individual contactors. This hypothesis can be tested by using vibration motors located at these predefined points to feedback finger positions. On the other hand, the movement of contactors was necessary for subjects to discriminate between objects of different sizes as shown in Experiment II. In this study, we did not explicitly control the amount of skin stretch with the movements of the contactors. Nevertheless, regarding the tactile acuity on the arm, the subjects might be utilizing perception during movement rather than the perceived location of the contactor. Therefore, the stretch of the skin might also help the subjects to some extent [15].

We only tested a limited set of object sizes in discrimination experiments. A wider range of object sizes can be tested to get a better estimate of subjective perceptions through the proposed sensory substitution device. In addition, we plan to conduct shape detection tests to further evaluate the efficacy of the method and the contribution of each feedback channel (i.e., motors of individual fingers).

We could not demonstrate the performance of the feedback method in a myoelectric robotic-hand control task. The main reason is the way we designed our study; we intended to test a method rather than propose a new sensory substitution device. As discussed in the previous section, implementation of such a method in myoelectric prosthesis requires major hardware changes (i.e., size) and software (i.e., delays in feedback). For example, the delay that we encountered in tasks with the glove is a major challenge for testing this method in a task where proportional control of robotic fingers is achieved with the aid of proposed feedback. Nevertheless, we foresee improving and testing this method in future works.

### Supplementary Information

Below is the link to the electronic supplementary material.

Supplementary file1 (PDF 1487 KB)

## Footnotes

1 The presumptive sensations that we cannot observe but are present in the model.
