# Supplementary material for: Three Sliding Probes Placed on Forelimb Skin for Proprioceptive Feedback Differentially yet Complementarily Contribute to Hand Gesture Detection and Object-Size Discrimination
Source: Ann Biomed Eng. 2024 Jan 21;52(4):982–96. doi: 10.1007/s10439-023-03434-4 (PMC10940487; doi:10.1007/s10439-023-03434-4)
Supplement: Supplementary file 1 — Supplementary file1 (PDF 1487 KB) [file 10439_2023_3434_MOESM1_ESM.pdf]

A Method to Provide Proprioceptive Information from Robotic Hands Through Sliding Probes on the Forelimb Skin

Supplementary Document

İsmail Devecioğlu and Ertuğrul Karakulak

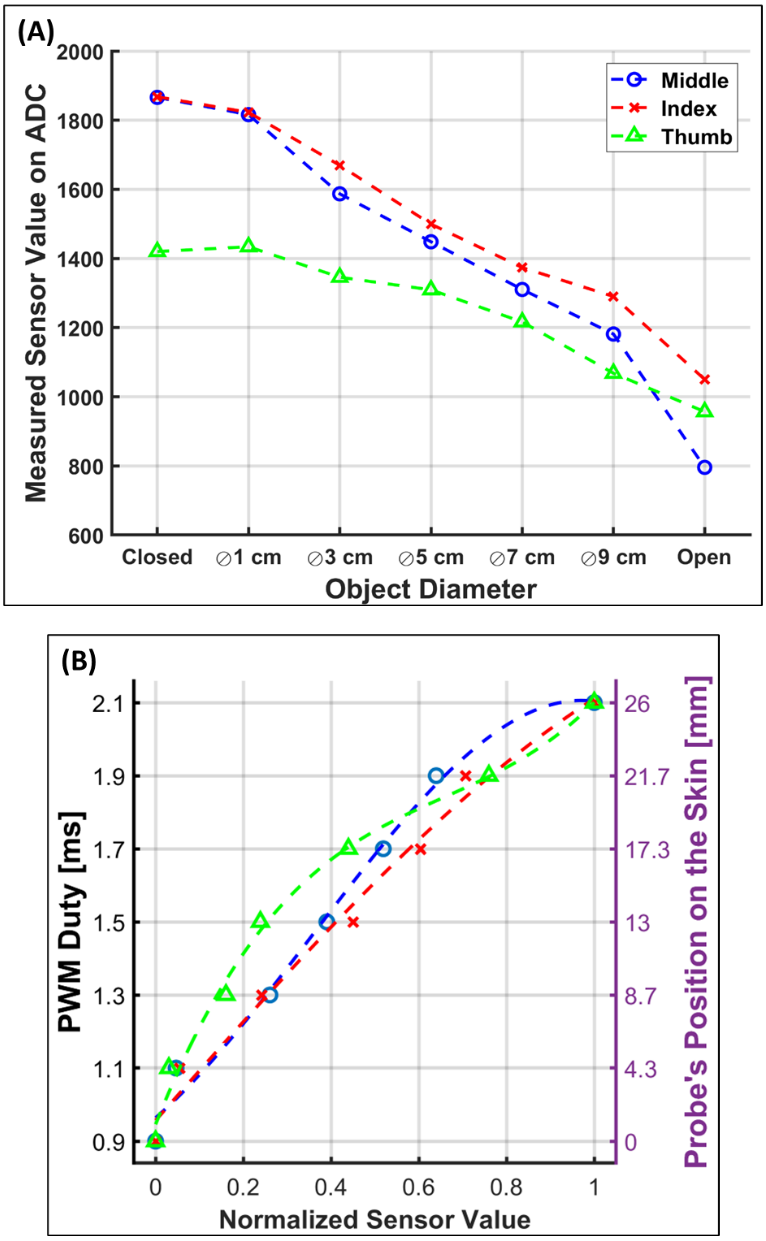

Fig. S1. Calibration of flex sensors on the glove. (A) Sensor output was recorded by the analog-to-digital converter (ADC) of the microcontroller while objects of different diameters were grasped or hand fully closed or opened. (B) Calibration curves that map tested objects and hand's open and closed states equidistantly on the skin.

Table S1. Summary of the leave-one-out SEM analysis for Experiment I. Estimation accuracy means the percentage of times the model correctly estimated the subject's response. BIC: the Bayesian information criterion, AIC: the Akaike information criterion. p-values >0.05 were colored in red.

(a) Volar condition

Subject	1	2	3	4	5	6	7	8	9	10	11	12	13	14	15	16	17	18	19	20	MEAN	STD
BIC	113.584	113.584	113.590	113.581	113.584	113.582	113.583	113.590	113.586	113.579	113.581	113.582	113.584	113.582	113.582	113.586	113.591	113.582	113.582	113.766	**113.593**	**0.040**
AIC	23.821	23.822	23.827	23.815	23.822	23.820	23.821	23.828	23.823	23.817	23.818	23.819	23.822	23.820	23.820	23.823	23.829	23.819	23.820	23.818	**23.821**	**0.003**
Estimation Accuracy	95.417	90.833	64.167	85.356	90.417	86.250	93.333	76.667	93.750	85.417	78.750	88.333	88.333	89.583	83.750	86.667	72.083	95.833	93.333	86.111	**86.219**	**7.858**
**ESTIMATES FOR ASSOCIATIONS**																						
Sthumb ~ Command_Gesture	0.114	0.115	0.114	0.114	0.114	0.114	0.114	0.114	0.114	0.114	0.115	0.114	0.114	0.114	0.114	0.114	0.115	0.114	0.114	0.114	**0.114**	**0.000**
Sindex ~ Command_Gesture	0.086	0.086	0.086	0.085	0.086	0.086	0.085	0.086	0.086	0.086	0.086	0.086	0.086	0.086	0.086	0.086	0.086	0.086	0.086	0.086	**0.086**	**0.000**
Smiddle□ Command_Gesture	0.200	0.200	0.200	0.200	0.199	0.200	0.199	0.200	0.200	0.200	0.200	0.200	0.200	0.200	0.200	0.200	0.200	0.200	0.200	0.200	**0.200**	**0.000**
P ~ Sthumb	1.827	1.827	1.838	1.838	1.835	1.834	1.834	1.834	1.834	1.834	1.835	1.835	1.835	1.834	1.834	1.833	1.837	1.826	1.826	1.835	**1.833**	**0.004**
P ~ Sindex	1.199	1.199	1.205	1.205	1.202	1.202	1.202	1.202	1.202	1.202	1.203	1.203	1.202	1.202	1.202	1.201	1.204	1.196	1.196	1.203	**1.202**	**0.002**
P ~ Smiddle	3.056	3.056	3.075	3.075	3.070	3.069	3.069	3.069	3.069	3.070	3.072	3.072	3.071	3.071	3.070	3.069	3.077	3.055	3.055	3.071	**3.068**	**0.007**
Thumb~Sthumb	1.000	1.000	1.000	1.000	1.000	1.000	1.000	1.000	1.000	1.000	1.000	1.000	1.000	1.000	1.000	1.000	1.000	1.000	1.000	1.000	**1.000**	**0.000**
Index~Sindex	1.000	1.000	1.000	1.000	1.000	1.000	1.000	1.000	1.000	1.000	1.000	1.000	1.000	1.000	1.000	1.000	1.000	1.000	1.000	1.000	**1.000**	**0.000**
Middle~Smiddle	1.000	1.000	1.000	1.000	1.000	1.000	1.000	1.000	1.000	1.000	1.000	1.000	1.000	1.000	1.000	1.000	1.000	1.000	1.000	1.000	**1.000**	**0.000**
Selected Gesture ~ P	1.000	1.000	1.000	1.000	1.000	1.000	1.000	1.000	1.000	1.000	1.000	1.000	1.000	1.000	1.000	1.000	1.000	1.000	1.000	1.000	**1.000**	**0.000**
P ~~ P	0.056	0.053	0.053	0.053	0.055	0.057	0.059	0.057	0.057	0.056	0.060	0.059	0.060	0.058	0.055	0.062	0.058	0.068	0.066	0.061	**0.058**	**0.004**
Sindex ~~ Sindex	0.011	0.012	0.006	0.009	0.012	0.012	0.011	0.008	0.012	0.010	0.012	0.011	0.010	0.010	0.013	0.010	0.009	0.012	0.011	0.013	**0.011**	**0.002**
Smiddle~~Smiddle	0.000	0.000	0.000	0.000	0.000	0.000	0.000	0.000	0.000	0.000	0.000	0.000	0.000	0.000	0.000	0.000	0.000	0.000	0.000	0.000	**0.000**	**0.000**
Sthumb~~Sthumb	0.013	0.013	0.012	0.012	0.012	0.011	0.013	0.012	0.013	0.011	0.011	0.012	0.012	0.013	0.013	0.012	0.012	0.013	0.013	0.013	**0.012**	**0.001**
Index~~Index	0.217	0.216	0.222	0.219	0.217	0.217	0.218	0.220	0.217	0.218	0.217	0.218	0.219	0.219	0.215	0.219	0.219	0.217	0.217	0.215	**0.218**	**0.002**
Middle~~Middle	0.133	0.133	0.133	0.133	0.133	0.133	0.133	0.133	0.133	0.133	0.133	0.133	0.133	0.133	0.133	0.133	0.133	0.133	0.133	0.133	**0.133**	**0.000**
Selected Gesture~~SelectedGesture	0.198	0.196	0.189	0.188	0.190	0.192	0.195	0.188	0.188	0.182	0.186	0.185	0.186	0.184	0.179	0.186	0.177	0.185	0.183	0.175	**0.187**	**0.006**
Thumb~~Thumb	0.171	0.171	0.172	0.172	0.172	0.173	0.171	0.172	0.171	0.173	0.173	0.172	0.172	0.171	0.171	0.172	0.172	0.171	0.171	0.171	**0.172**	**0.001**
**p-vals for above estimations**																						
Sthumb ~ Command_Gesture	0.000	0.000	0.000	0.000	0.000	0.000	0.000	0.000	0.000	0.000	0.000	0.000	0.000	0.000	0.000	0.000	0.000	0.000	0.000	0.000		
Sindex ~ Command_Gesture	0.000	0.000	0.000	0.000	0.000	0.000	0.000	0.000	0.000	0.000	0.000	0.000	0.000	0.000	0.000	0.000	0.000	0.000	0.000	0.000		
Smiddle□ Command_Gesture	0.000	0.000	0.000	0.000	0.000	0.000	0.000	0.000	0.000	0.000	0.000	0.000	0.000	0.000	0.000	0.000	0.000	0.000	0.000	0.000		
P ~ Sthumb	0.000	0.000	0.000	0.000	0.000	0.000	0.000	0.000	0.000	0.000	0.000	0.000	0.000	0.000	0.000	0.000	0.000	0.000	0.000	0.000		
P ~ Sindex	0.000	0.000	0.000	0.000	0.000	0.000	0.000	0.000	0.000	0.000	0.000	0.000	0.000	0.000	0.000	0.000	0.000	0.000	0.000	0.000		
P ~ Smiddle	0.000	0.000	0.000	0.000	0.000	0.000	0.000	0.000	0.000	0.000	0.000	0.000	0.000	0.000	0.000	0.000	0.000	0.000	0.000	0.000		
Thumb~Sthumb	-	-	-	-	-	-	-	-	-	-	-	-	-	-	-	-	-	-	-	-		
Index~Sindex	-	-	-	-	-	-	-	-	-	-	-	-	-	-	-	-	-	-	-	-		
Middle~Smiddle	-	-	-	-	-	-	-	-	-	-	-	-	-	-	-	-	-	-	-	-		
Selected Gesture ~ P	-	-	-	-	-	-	-	-	-	-	-	-	-	-	-	-	-	-	-	-		
P ~~ P	0.000	0.000	0.000	0.000	0.000	0.000	0.000	0.000	0.000	0.000	0.000	0.000	0.000	0.000	0.000	0.000	0.000	0.000	0.000	0.000		
Sindex ~~ Sindex	0.001	0.000	0.041	0.004	0.000	0.000	0.001	0.011	0.000	0.001	0.000	0.001	0.002	0.002	0.000	0.003	0.003	0.000	0.001	0.000		
Smiddle~~Smiddle	1.000	1.000	1.000	1.000	1.000	1.000	1.000	1.000	1.000	1.000	1.000	1.000	1.000	1.000	1.000	0.996	0.998	1.000	1.000	1.000		
Sthumb~~Sthumb	0.000	0.000	0.000	0.000	0.000	0.000	0.000	0.000	0.000	0.000	0.000	0.000	0.000	0.000	0.000	0.000	0.000	0.000	0.000	0.000		
Index~~Index	0.000	0.000	0.000	0.000	0.000	0.000	0.000	0.000	0.000	0.000	0.000	0.000	0.000	0.000	0.000	0.000	0.000	0.000	0.000	0.000		
Middle~~Middle	0.000	0.000	0.000	0.000	0.000	0.000	0.000	0.000	0.000	0.000	0.000	0.000	0.000	0.000	0.000	0.000	0.000	0.000	0.000	0.000		
Selected Gesture~~SelectedGesture	0.000	0.000	0.000	0.000	0.000	0.000	0.000	0.000	0.000	0.000	0.000	0.000	0.000	0.000	0.000	0.000	0.000	0.000	0.000	0.000		
Thumb~~Thumb	0.000	0.000	0.000	0.000	0.000	0.000	0.000	0.000	0.000	0.000	0.000	0.000	0.000	0.000	0.000	0.000	0.000	0.000	0.000	0.000		

(b) Dorsal condition

SID	1	2	3	4	5	6	7	8	9	10	11	12	13	14	15	16	17	18	19	20	MEAN	STD
BIC	113.732	113.727	113.728	113.728	113.727	113.723	113.726	113.727	113.721	113.718	113.738	113.726	113.723	113.730	113.724	113.729	113.728	113.730	113.723	113.727	**113.727**	0.004
AIC	23.802	23.794	23.798	23.795	23.797	23.793	23.796	23.797	23.789	23.788	23.808	23.793	23.788	23.801	23.794	23.800	23.798	23.800	23.794	23.795	**23.796**	0.005
Estimation Accuracy	59.583	80.753	72.500	76.987	62.500	71.250	62.917	69.167	77.824	82.500	75.833	71.548	76.050	52.500	76.250	67.083	64.583	62.083	77.917	82.427	**71.113**	8.113
**ESTIMATES FOR ASSOCIATIONS**																						
Sthumb ~ Command_Gesture	0.114	0.115	0.115	0.114	0.114	0.114	0.114	0.115	0.114	0.114	0.114	0.114	0.114	0.114	0.114	0.114	0.114	0.114	0.114	0.114	**0.114**	0.000
Sindex ~ Command_Gesture	0.086	0.086	0.086	0.086	0.086	0.086	0.086	0.086	0.086	0.085	0.085	0.086	0.086	0.086	0.086	0.086	0.086	0.086	0.086	0.086	**0.086**	0.000
Smiddle� Command_Gesture	0.200	0.201	0.201	0.200	0.200	0.200	0.200	0.200	0.200	0.199	0.199	0.200	0.200	0.200	0.200	0.199	0.200	0.200	0.200	0.200	**0.200**	0.000
P ~ Sthumb	1.809	1.809	1.809	1.809	1.809	1.809	1.809	1.811	1.821	1.820	1.820	1.811	1.812	1.811	1.811	1.811	1.811	1.810	1.810	1.814	**1.812**	0.004
P ~ Sindex	1.225	1.225	1.225	1.225	1.226	1.225	1.225	1.226	1.234	1.233	1.233	1.226	1.227	1.226	1.226	1.226	1.226	1.225	1.225	1.228	**1.227**	0.003
P ~ Smiddle	3.034	3.034	3.034	3.034	3.035	3.035	3.035	3.039	3.057	3.056	3.055	3.041	3.045	3.043	3.042	3.042	3.042	3.040	3.040	3.049	**3.042**	0.007
Thumb~Sthumb	1.000	1.000	1.000	1.000	1.000	1.000	1.000	1.000	1.000	1.000	1.000	1.000	1.000	1.000	1.000	1.000	1.000	1.000	1.000	1.000	**1.000**	0.000
Index~Sindex	1.000	1.000	1.000	1.000	1.000	1.000	1.000	1.000	1.000	1.000	1.000	1.000	1.000	1.000	1.000	1.000	1.000	1.000	1.000	1.000	**1.000**	0.000
Middle~Smiddle	1.000	1.000	1.000	1.000	1.000	1.000	1.000	1.000	1.000	1.000	1.000	1.000	1.000	1.000	1.000	1.000	1.000	1.000	1.000	1.000	**1.000**	0.000
Selected Gesture ~ P	1.000	1.000	1.000	1.000	1.000	1.000	1.000	1.000	1.000	1.000	1.000	1.000	1.000	1.000	1.000	1.000	1.000	1.000	1.000	1.000	**1.000**	0.000
P ~~ P	0.059	0.055	0.053	0.057	0.057	0.058	0.060	0.056	0.052	0.052	0.057	0.059	0.054	0.065	0.067	0.068	0.063	0.066	0.064	0.058	**0.059**	0.005
Sindex ~~ Sindex	0.021	0.023	0.022	0.025	0.022	0.025	0.023	0.021	0.024	0.022	0.020	0.023	0.022	0.020	0.023	0.022	0.024	0.022	0.024	0.025	**0.023**	0.001
Smiddle~~Smiddle	0.000	0.000	0.000	0.000	0.000	0.000	0.000	0.000	0.000	0.000	0.000	0.000	0.000	0.000	0.000	0.000	0.000	0.000	0.000	0.000	**0.000**	0.000
Sthumb~~Sthumb	0.012	0.012	0.013	0.013	0.011	0.013	0.013	0.013	0.013	0.014	0.011	0.013	0.014	0.013	0.013	0.013	0.012	0.013	0.013	0.014	**0.013**	0.001
Index~~Index	0.207	0.206	0.207	0.204	0.207	0.204	0.205	0.208	0.205	0.206	0.208	0.205	0.206	0.209	0.205	0.206	0.205	0.206	0.205	0.203	**0.206**	0.001
Middle~~Middle	0.133	0.133	0.133	0.133	0.133	0.133	0.133	0.133	0.133	0.133	0.133	0.133	0.133	0.133	0.133	0.133	0.133	0.133	0.133	0.133	**0.133**	0.000
Selected Gesture~~SelectedGesture	0.254	0.251	0.248	0.252	0.252	0.253	0.255	0.246	0.242	0.242	0.246	0.248	0.232	0.243	0.245	0.246	0.240	0.243	0.240	0.226	**0.245**	0.007
Thumb~~Thumb	0.172	0.172	0.171	0.171	0.173	0.172	0.171	0.172	0.171	0.171	0.174	0.172	0.170	0.171	0.171	0.171	0.172	0.172	0.171	0.170	**0.171**	0.001
**p-vals for above estimations**																						
Sthumb ~ Command_Gesture	0.000	0.000	0.000	0.000	0.000	0.000	0.000	0.000	0.000	0.000	0.000	0.000	0.000	0.000	0.000	0.000	0.000	0.000	0.000	0.000		
Sindex ~ Command_Gesture	0.000	0.000	0.000	0.000	0.000	0.000	0.000	0.000	0.000	0.000	0.000	0.000	0.000	0.000	0.000	0.000	0.000	0.000	0.000	0.000		
Smiddle� Command_Gesture	0.000	0.000	0.000	0.000	0.000	0.000	0.000	0.000	0.000	0.000	0.000	0.000	0.000	0.000	0.000	0.000	0.000	0.000	0.000	0.000		
P ~ Sthumb	0.000	0.000	0.000	0.000	0.000	0.000	0.000	0.000	0.000	0.000	0.000	0.000	0.000	0.000	0.000	0.000	0.000	0.000	0.000	1.000		
P ~ Sindex	0.000	0.000	0.000	0.000	0.000	0.000	0.000	0.000	0.000	0.000	0.000	0.000	0.000	0.000	0.000	0.000	0.000	0.000	0.000	1.000		
P ~ Smiddle	0.000	0.000	0.000	0.000	0.000	0.000	0.000	0.000	0.000	0.000	0.000	0.000	0.000	0.000	0.000	0.000	0.000	0.000	0.000	1.000		
Thumb~Sthumb	-	-	-	-	-	-	-	-	-	-	-	-	-	-	-	-	-	-	-	-		
Index~Sindex	-	-	-	-	-	-	-	-	-	-	-	-	-	-	-	-	-	-	-	-		
Middle~Smiddle	-	-	-	-	-	-	-	-	-	-	-	-	-	-	-	-	-	-	-	-		
Selected Gesture ~ P	-	-	-	-	-	-	-	-	-	-	-	-	-	-	-	-	-	-	-	-		
P ~~ P	0.000	0.000	0.000	0.000	0.000	0.000	0.000	0.000	0.000	0.000	0.000	0.000	0.000	0.000	0.000	0.000	0.000	0.000	0.000	1.000		
Sindex ~~ Sindex	0.000	0.000	0.000	0.000	0.000	0.000	0.000	0.000	0.000	0.000	0.000	0.000	0.000	0.000	0.000	0.000	0.000	0.000	0.000	1.000		
Smiddle~~Smiddle	1.000	1.000	1.000	1.000	1.000	1.000	1.000	1.000	1.000	1.000	1.000	1.000	1.000	1.000	1.000	1.000	1.000	1.000	1.000	1.000		
Sthumb~~Sthumb	0.000	0.000	0.000	0.000	0.000	0.000	0.000	0.000	0.000	0.000	0.000	0.000	0.000	0.000	0.000	0.000	0.000	0.000	0.000	1.000		
Index~~Index	0.000	0.000	0.000	0.000	0.000	0.000	0.000	0.000	0.000	0.000	0.000	0.000	0.000	0.000	0.000	0.000	0.000	0.000	0.000	1.000		
Middle~~Middle	0.000	0.000	0.000	0.000	0.000	0.000	0.000	0.000	0.000	0.000	0.000	0.000	0.000	0.000	0.000	0.000	0.000	0.000	0.000	0.000		
Selected Gesture~~SelectedGesture	0.000	0.000	0.000	0.000	0.000	0.000	0.000	0.000	0.000	0.000	0.000	0.000	0.000	0.000	0.000	0.000	0.000	0.000	0.000	1.000		
Thumb~~Thumb	0.000	0.000	0.000	0.000	0.000	0.000	0.000	0.000	0.000	0.000	0.000	0.000	0.000	0.000	0.000	0.000	0.000	0.000	0.000	1.000		

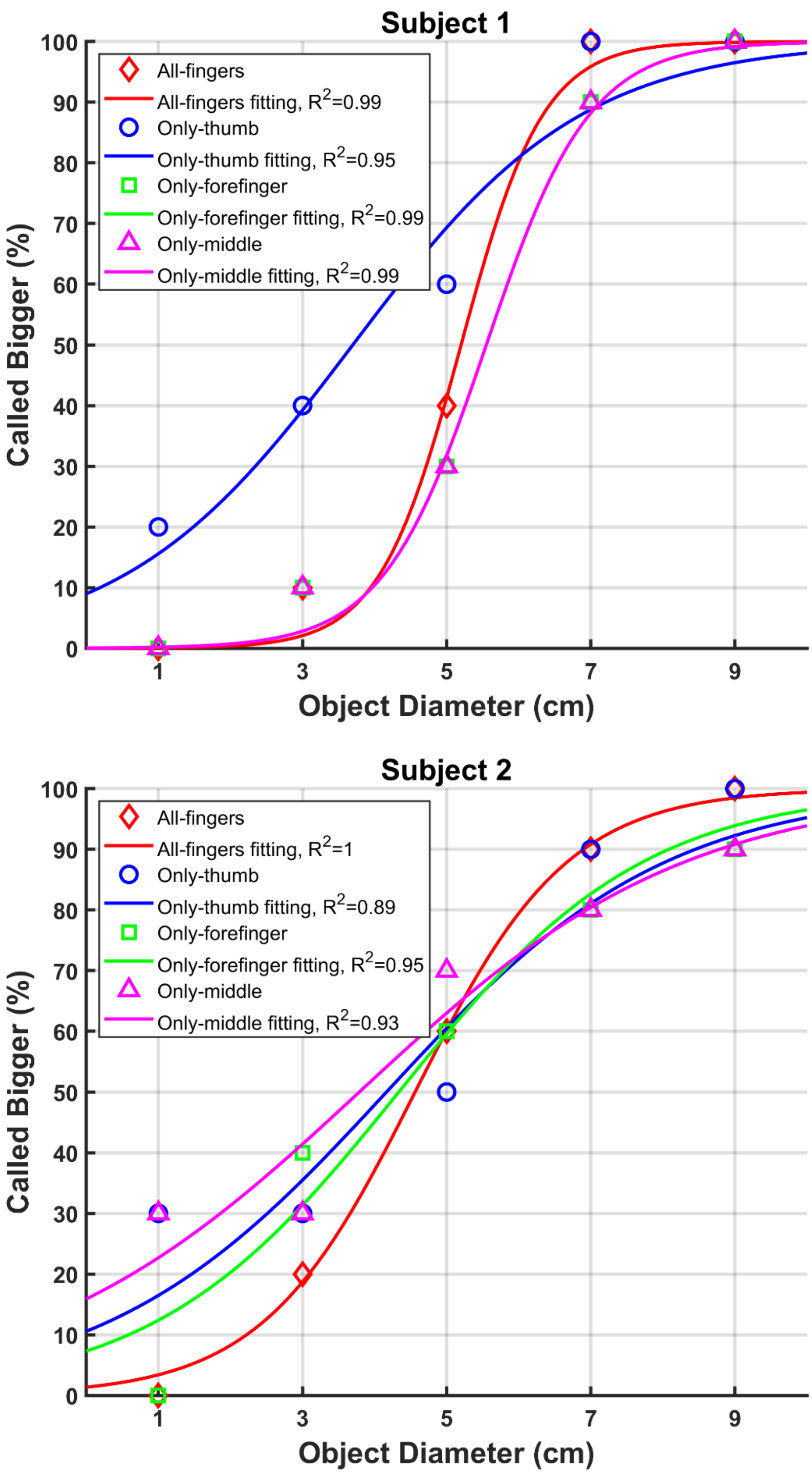

Fig. S2. Object size discrimination performance of each subject. The data for each condition was fitted with Eq. (2), and R^2 values of the fits are given in the legend.

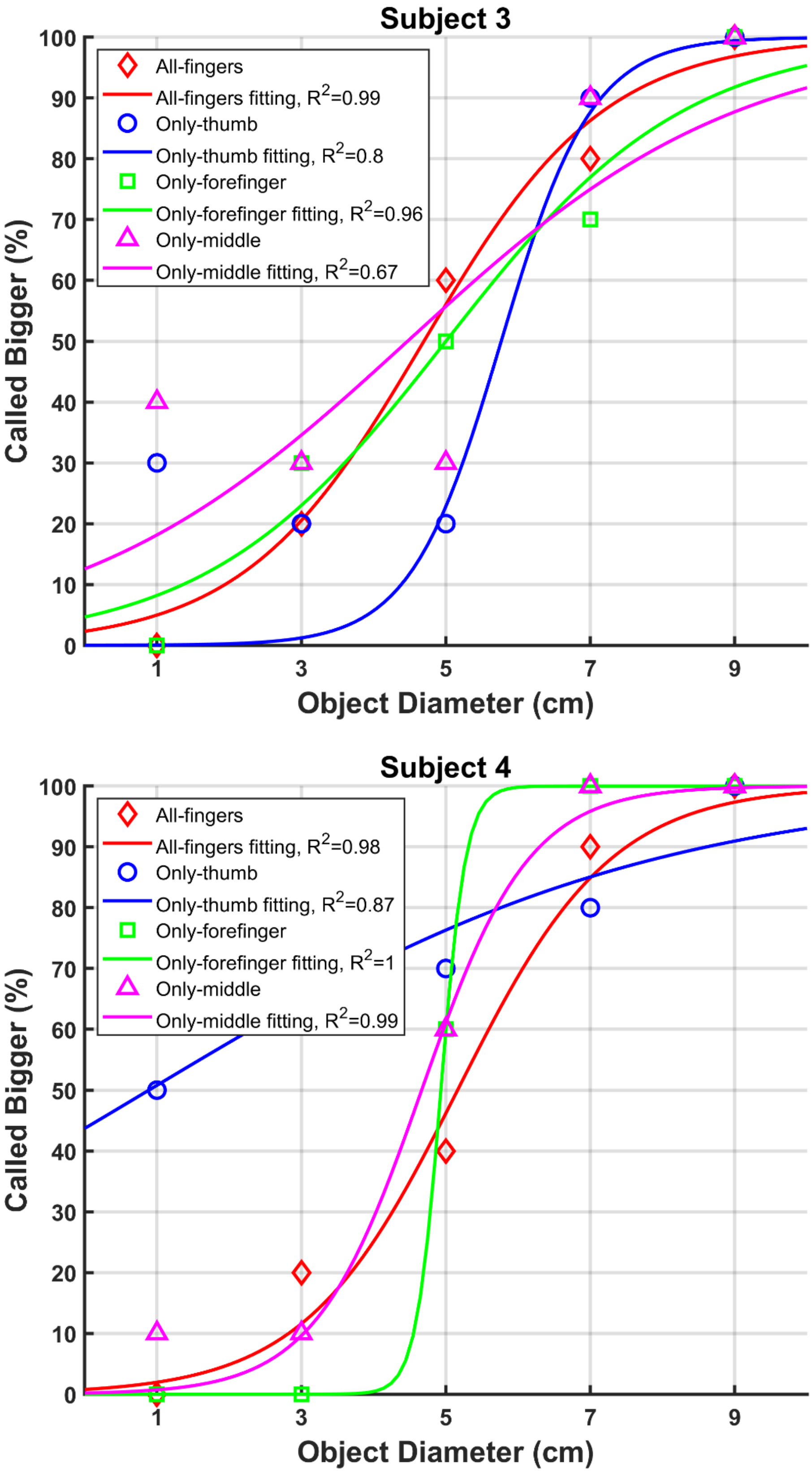

Fig. S2. Continued.

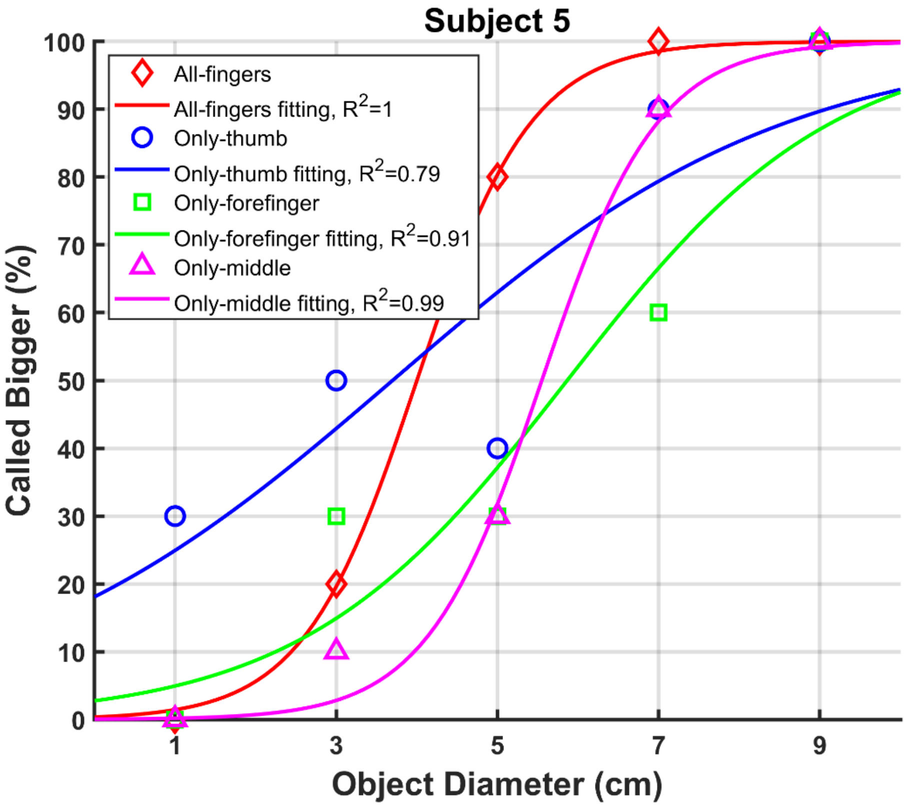

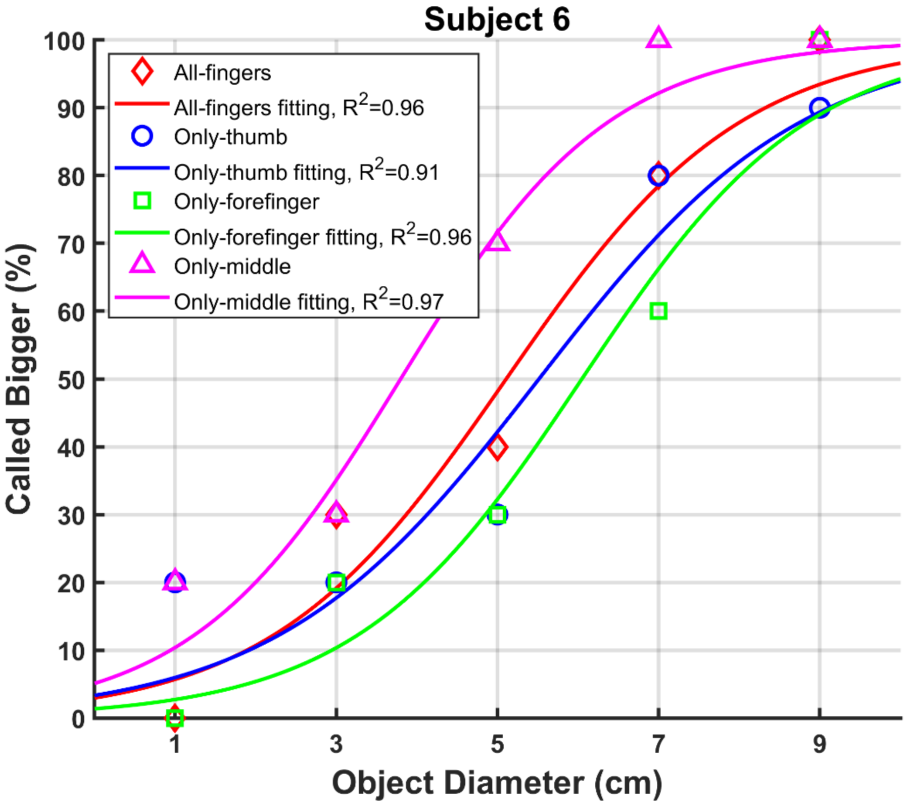

Fig. S2. Continued.

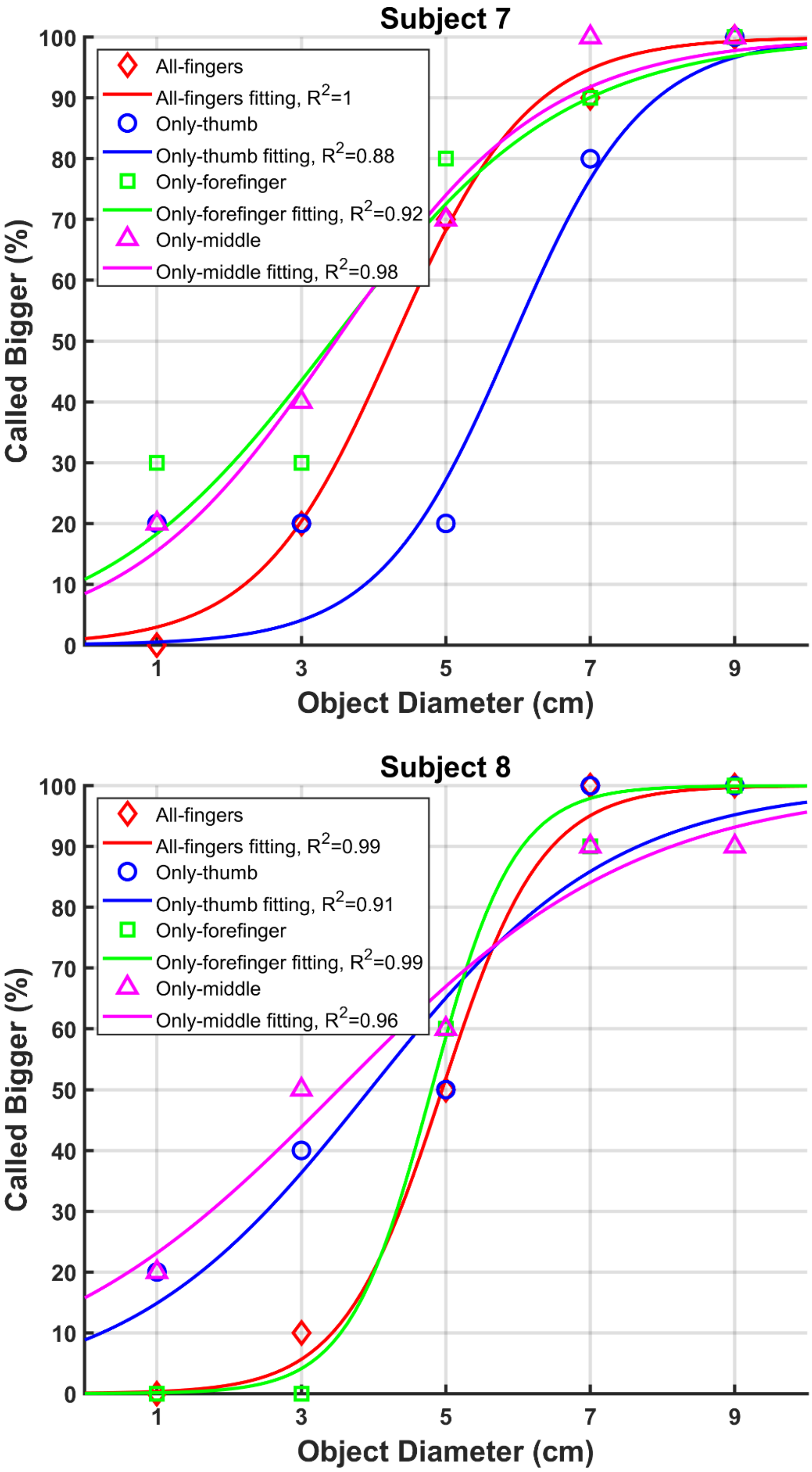

Fig. S2. Continued.

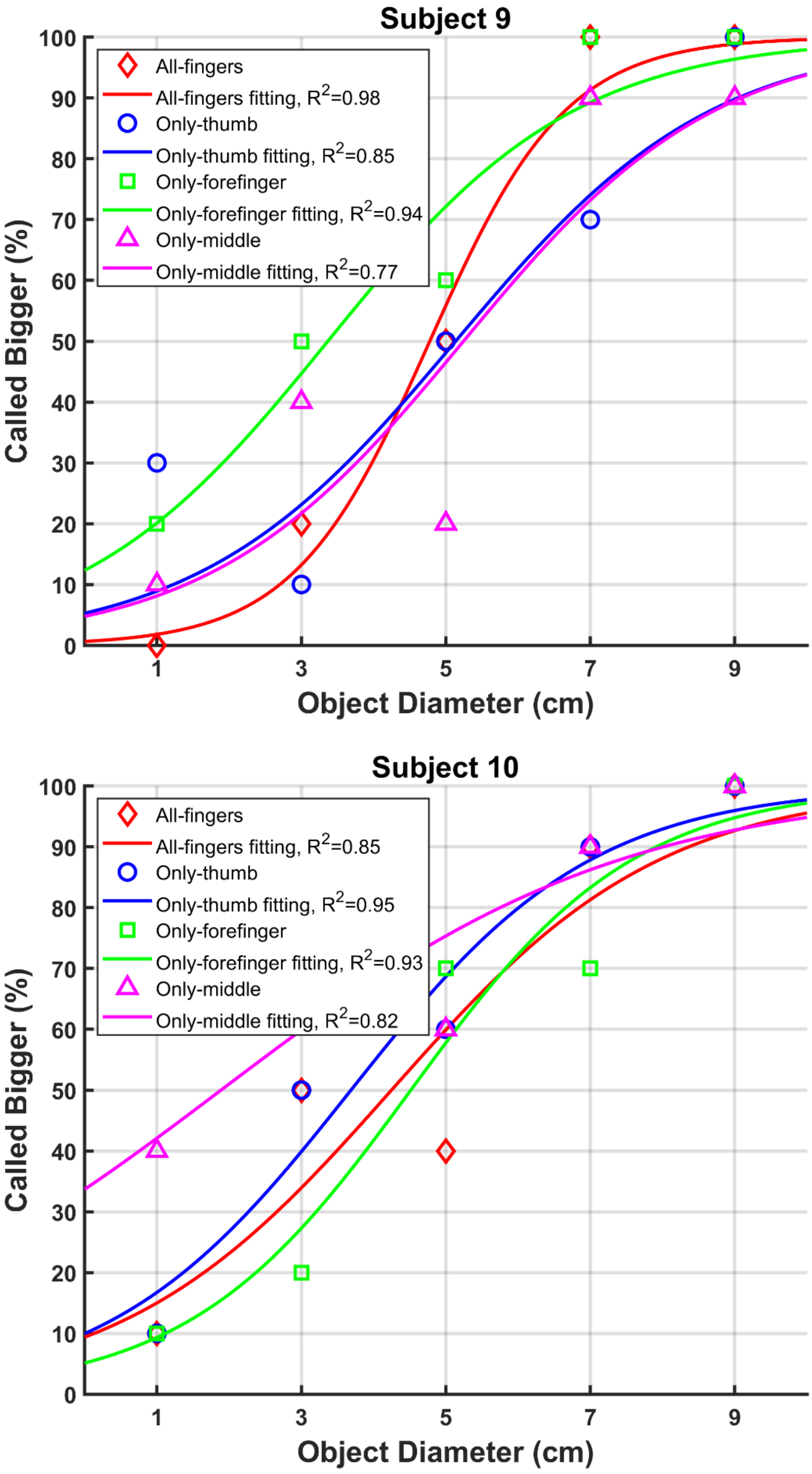

Fig. S2. Continued.

Table S2. Summary of the leave-one-out SEM analysis for Experiment II. Estimation accuracy means the percentage of times the model correctly estimated the subject’s response. BIC: the Bayesian information criterion, AIC: the Akaike information criterion. p-values >0.05 were colored in red.

Subject			1	2	3	4	5	6	7	8	9	10	MEAN	SD
**BIC**			223.862	223.864	223.862	223.865	223.856	223.861	223.861	223.860	223.865	223.862	**223.862**	**0.003**
**AIC**			55.146	55.147	55.145	55.149	55.139	55.144	55.144	55.143	55.148	55.145	**55.145**	**0.003**
**Model Accuracy**			85.000	86.250	85.625	80.000	89.375	83.750	85.625	87.500	93.125	88.125	**86.438**	**3.487**
**ESTIMATES FOR ASSOCIATIONS**														
**Sthumb1**	~	**Stimulus_atInterval1**	2.209	2.309	2.192	2.147	2.203	2.163	2.139	2.242	2.189	2.244	**2.204**	**0.051**
**Sthumb2**	~	**Stimulus_atInterval2**	2.006	1.914	2.023	2.082	2.029	2.050	2.072	2.015	2.071	2.031	**2.029**	**0.048**
**Sindex1**	~	**Stimulus_atInterval1**	2.191	2.204	2.165	2.126	2.185	2.186	2.173	2.129	2.118	2.060	**2.154**	**0.045**
**Sindex2**	~	**Stimulus_atInterval2**	2.363	2.350	2.405	2.413	2.348	2.378	2.384	2.376	2.352	2.379	**2.375**	**0.022**
**Smiddle1**	~	**Stimulus_atInterval1**	2.128	2.172	2.087	2.082	2.098	2.083	2.113	2.181	2.164	2.145	**2.125**	**0.038**
**Smiddle2**	~	**Stimulus_atInterval2**	2.161	2.090	2.178	2.192	2.178	2.200	2.167	2.073	2.136	2.137	**2.151**	**0.042**
**P**	~	**Sthumb1**	-0.010	-0.007	-0.012	-0.008	-0.008	-0.011	-0.021	-0.026	-0.021	-0.039	**-0.016**	**0.010**
**P**	~	**Sindex1**	-0.133	-0.141	-0.139	-0.127	-0.125	-0.130	-0.131	-0.144	-0.127	-0.124	**-0.132**	**0.007**
**P**	~	**Smiddle1**	-0.067	-0.057	-0.061	-0.080	-0.074	-0.069	-0.059	-0.039	-0.058	-0.048	**-0.061**	**0.012**
**P**	~	**Sthumb2**	0.018	0.014	0.018	0.010	0.020	0.016	0.023	0.024	0.023	0.042	**0.021**	**0.009**
**P**	~	**Sindex2**	0.127	0.144	0.135	0.132	0.123	0.133	0.131	0.137	0.141	0.126	**0.133**	**0.007**
**P**	~	**Smiddle2**	0.062	0.053	0.051	0.065	0.065	0.059	0.053	0.046	0.043	0.043	**0.054**	**0.009**
**ThumbPosition_atInterval1**	~	**Sthumb1**	1.000	1.000	1.000	1.000	1.000	1.000	1.000	1.000	1.000	1.000	**1.000**	**0.000**
**ThumbPosition_atInterval2**	~	**Sthumb2**	1.000	1.000	1.000	1.000	1.000	1.000	1.000	1.000	1.000	1.000	**1.000**	**0.000**
**IndexPosition_atInterval1**	~	**Sindex1**	1.000	1.000	1.000	1.000	1.000	1.000	1.000	1.000	1.000	1.000	**1.000**	**0.000**
**IndexPosition_atInterval2**	~	**Sindex2**	1.000	1.000	1.000	1.000	1.000	1.000	1.000	1.000	1.000	1.000	**1.000**	**0.000**
**MiddlePosition_atInterval1**	~	**Smiddle1**	1.000	1.000	1.000	1.000	1.000	1.000	1.000	1.000	1.000	1.000	**1.000**	**0.000**
**MiddlePosition_atInterval2**	~	**Smiddle2**	1.000	1.000	1.000	1.000	1.000	1.000	1.000	1.000	1.000	1.000	**1.000**	**0.000**
**Selected_Interval**	~	**P**	1.000	1.000	1.000	1.000	1.000	1.000	1.000	1.000	1.000	1.000	**1.000**	**0.000**
**P**	~~	**P**	0.145	0.101	0.110	0.099	0.123	0.106	0.117	0.111	0.126	0.127	**0.116**	**0.014**
**Sindex1**	~~	**Sindex1**	9.399	9.404	9.375	9.350	9.363	9.336	9.332	9.120	9.386	9.417	**9.348**	**0.085**
**Sindex2**	~~	**Sindex2**	7.975	7.947	7.955	7.971	7.941	7.944	7.948	7.936	7.673	7.605	**7.889**	**0.134**
**Smiddle1**	~~	**Smiddle1**	9.872	9.855	9.819	9.795	9.779	9.739	9.735	9.128	9.575	9.581	**9.688**	**0.221**
**Smiddle2**	~~	**Smiddle2**	9.762	9.762	9.783	9.787	9.784	9.796	9.798	10.286	9.761	9.746	**9.827**	**0.162**
**Sthumb1**	~~	**Sthumb1**	8.869	8.867	8.854	8.874	8.878	8.888	8.889	8.655	8.815	8.826	**8.842**	**0.070**
**Sthumb2**	~~	**Sthumb2**	10.038	10.042	10.055	10.042	10.036	10.027	10.026	10.155	9.987	9.964	**10.037**	**0.050**
**ThumbPosition_atInterval1**	~~	**Stimulus_atInterval1**	-0.218	-0.232	-0.148	-0.117	-0.209	-0.106	-0.127	-0.154	-0.209	-0.240	**-0.176**	**0.051**
**ThumbPosition_atInterval1**	~~	**ThumbPosition_atInterval1**	37.955	37.952	37.942	37.965	37.969	37.985	37.987	37.765	37.940	37.940	**37.940**	**0.064**
**ThumbPosition_atInterval2**	~~	**Stimulus_atInterval2**	0.370	0.376	0.299	0.249	0.344	0.262	0.274	0.247	0.297	0.319	**0.304**	**0.048**
**ThumbPosition_atInterval2**	~~	**ThumbPosition_atInterval2**	39.863	39.870	39.884	39.871	39.869	39.860	39.859	40.062	39.922	39.918	**39.898**	**0.062**
**IndexPosition_atInterval1**	~~	**Stimulus_atInterval1**	0.167	0.148	0.179	0.150	0.230	0.167	0.160	0.206	0.200	0.287	**0.189**	**0.043**
**IndexPosition_atInterval1**	~~	**IndexPosition_atInterval1**	38.898	38.898	38.901	38.917	38.930	38.964	38.966	38.816	39.168	39.171	**38.963**	**0.117**
**IndexPosition_atInterval2**	~~	**Stimulus_atInterval2**	-0.419	-0.399	-0.441	-0.391	-0.462	-0.427	-0.420	-0.406	-0.359	-0.409	**-0.413**	**0.028**
**IndexPosition_atInterval2**	~~	**IndexPosition_atInterval2**	38.525	38.550	38.570	38.575	38.591	38.591	38.591	39.188	39.178	39.197	**38.755**	**0.299**
**MiddlePosition_atInterval1**	~~	**Stimulus_atInterval1**	0.053	0.018	0.050	0.075	0.021	0.005	0.044	-0.080	0.040	-0.025	**0.020**	**0.045**
**MiddlePosition_atInterval1**	~~	**MiddlePosition_atInterval1**	38.299	38.281	38.257	38.258	38.242	38.232	38.231	37.642	38.127	38.122	**38.169**	**0.194**
**MiddlePosition_atInterval2**	~~	**Stimulus_atInterval2**	0.020	0.078	0.043	0.004	0.063	0.066	0.027	0.175	0.006	0.092	**0.058**	**0.051**
**MiddlePosition_atInterval2**	~~	**MiddlePosition_atInterval2**	38.785	38.799	38.824	38.824	38.838	38.849	38.851	39.481	39.012	39.018	**38.928**	**0.211**
**Selected_Interval**	~~	**Selected_Interval**	0.000	0.019	0.027	0.022	0.045	0.034	0.045	0.039	0.054	0.059	**0.034**	**0.018**
**P-vals for above estimations in the same order**														
**Sthumb1**	~	**Stimulus_atInterval1**	0.000	0.000	0.000	0.000	0.000	0.000	0.000	0.000	0.000	0.000		
**Sthumb2**	~	**Stimulus_atInterval2**	0.000	0.000	0.000	0.000	0.000	0.000	0.000	0.000	0.000	0.000		
**Sindex1**	~	**Stimulus_atInterval1**	0.000	0.000	0.000	0.000	0.000	0.000	0.000	0.000	0.000	0.000		
**Sindex2**	~	**Stimulus_atInterval2**	0.000	0.000	0.000	0.000	0.000	0.000	0.000	0.000	0.000	0.000		
**Smiddle1**	~	**Stimulus_atInterval1**	0.000	0.000	0.000	0.000	0.000	0.000	0.000	0.000	0.000	0.000		
**Smiddle2**	~	**Stimulus_atInterval2**	0.000	0.000	0.000	0.000	0.000	0.000	0.000	0.000	0.000	0.000		
**P**	~	**Sthumb1**	0.686	0.798	0.640	0.749	0.741	0.658	0.394	0.277	0.391	0.068		
**P**	~	**Sindex1**	0.001	0.001	0.001	0.001	0.001	0.001	0.001	0.001	0.001	0.000		
**P**	~	**Smiddle1**	0.000	0.002	0.001	0.000	0.000	0.000	0.002	0.058	0.002	0.014		
**P**	~	**Sthumb2**	0.402	0.533	0.404	0.664	0.361	0.467	0.282	0.266	0.305	0.037		
**P**	~	**Sindex2**	0.000	0.001	0.000	0.000	0.000	0.000	0.000	0.001	0.001	0.000		
**P**	~	**Smiddle2**	0.001	0.011	0.010	0.001	0.001	0.003	0.007	0.022	0.040	0.044		
**ThumbPosition_atInterval1**	~	**Sthumb1**	-	-	-	-	-	-	-	-	-	-		
**ThumbPosition_atInterval2**	~	**Sthumb2**	-	-	-	-	-	-	-	-	-	-		
**IndexPosition_atInterval1**	~	**Sindex1**	-	-	-	-	-	-	-	-	-	-		
**IndexPosition_atInterval2**	~	**Sindex2**	-	-	-	-	-	-	-	-	-	-		
**MiddlePosition_atInterval1**	~	**Smiddle1**	-	-	-	-	-	-	-	-	-	-		
**MiddlePosition_atInterval2**	~	**Smiddle2**	-	-	-	-	-	-	-	-	-	-		
**Selected_Interval**	~	**P**	-	-	-	-	-	-	-	-	-	-		
**P**	~~	**P**	0.002	0.065	0.029	0.036	0.005	0.022	0.008	0.026	0.006	0.001		
**Sindex1**	~~	**Sindex1**	0.000	0.000	0.000	0.000	0.000	0.000	0.000	0.000	0.000	0.000		
**Sindex2**	~~	**Sindex2**	0.000	0.000	0.000	0.000	0.000	0.000	0.000	0.000	0.000	0.000		
**Smiddle1**	~~	**Smiddle1**	0.000	0.000	0.000	0.000	0.000	0.000	0.000	0.000	0.000	0.000		
**Smiddle2**	~~	**Smiddle2**	0.000	0.000	0.000	0.000	0.000	0.000	0.000	0.000	0.000	0.000		
**Sthumb1**	~~	**Sthumb1**	0.000	0.000	0.000	0.000	0.000	0.000	0.000	0.000	0.000	0.000		
**Sthumb2**	~~	**Sthumb2**	0.000	0.000	0.000	0.000	0.000	0.000	0.000	0.000	0.000	0.000		
**ThumbPosition_atInterval1**	~~	**Stimulus_atInterval1**	0.667	0.646	0.774	0.821	0.682	0.836	0.801	0.759	0.681	0.648		
**ThumbPosition_atInterval1**	~~	**ThumbPosition_atInterval1**	0.000	0.000	0.000	0.000	0.000	0.000	0.000	0.000	0.000	0.000		
**ThumbPosition_atInterval2**	~~	**Stimulus_atInterval2**	0.439	0.437	0.527	0.598	0.473	0.580	0.567	0.618	0.543	0.507		
**ThumbPosition_atInterval2**	~~	**ThumbPosition_atInterval2**	0.000	0.000	0.000	0.000	0.000	0.000	0.000	0.000	0.000	0.000		
**IndexPosition_atInterval1**	~~	**Stimulus_atInterval1**	0.742	0.761	0.728	0.772	0.654	0.749	0.757	0.670	0.688	0.559		
**IndexPosition_atInterval1**	~~	**IndexPosition_atInterval1**	0.000	0.000	0.000	0.000	0.000	0.000	0.000	0.000	0.000	0.000		
**IndexPosition_atInterval2**	~~	**Stimulus_atInterval2**	0.446	0.493	0.422	0.467	0.394	0.428	0.437	0.479	0.510	0.460		
**IndexPosition_atInterval2**	~~	**IndexPosition_atInterval2**	0.000	0.000	0.000	0.000	0.000	0.000	0.000	0.000	0.000	0.000		
**MiddlePosition_atInterval1**	~~	**Stimulus_atInterval1**	0.915	0.969	0.919	0.882	0.965	0.992	0.929	0.870	0.937	0.960		
**MiddlePosition_atInterval1**	~~	**MiddlePosition_atInterval1**	0.000	0.000	0.000	0.000	0.000	0.000	0.000	0.000	0.000	0.000		
**MiddlePosition_atInterval2**	~~	**Stimulus_atInterval2**	0.968	0.881	0.932	0.994	0.901	0.896	0.956	0.731	0.990	0.854		
**MiddlePosition_atInterval2**	~~	**MiddlePosition_atInterval2**	0.000	0.000	0.000	0.000	0.000	0.000	0.000	0.000	0.000	0.000		
**Selected_Interval**	~~	**Selected_Interval**	1.000	0.732	0.586	0.646	0.296	0.465	0.308	0.438	0.241	0.113		
